# Current understanding of the molecular mechanisms in Parkinson's disease: Targets for potential treatments

**DOI:** 10.1186/s40035-017-0099-z

**Published:** 2017-10-25

**Authors:** Panchanan Maiti, Jayeeta Manna, Gary L. Dunbar

**Affiliations:** 1 Field Neurosciences Institute Laboratory for Restorative Neurology, Mt. Pleasant, MI 48859 USA; 2 Program in Neuroscience, Mt. Pleasant, MI 48859 USA; 3 Department of Psychology, Central Michigan University, Mt. Pleasant, MI 48859 USA; 4 Field Neurosciences Institute, St. Mary’s of Michigan, Saginaw, MI 48604 USA; 5 Department of Biology, Saginaw Valley State University, Saginaw, MI 48604 USA; 6 Department of Physiology, University of Tennessee Health Science Center, Memphis, TN 38105 USA

**Keywords:** Parkinson’s disease, Neurodegeneration, Protein misfolding, Molecular chaperones, Cell therapy

## Abstract

Gradual degeneration and loss of dopaminergic neurons in the substantia nigra, pars compacta and subsequent reduction of dopamine levels in striatum are associated with motor deficits that characterize Parkinson’s disease (PD). In addition, half of the PD patients also exhibit frontostriatal-mediated executive dysfunction, including deficits in attention, short-term working memory, speed of mental processing, and impulsivity. The most commonly used treatments for PD are only partially or transiently effective and are available or applicable to a minority of patients. Because, these therapies neither restore the lost or degenerated dopaminergic neurons, nor prevent or delay the disease progression, the need for more effective therapeutics is critical. In this review, we provide a comprehensive overview of the current understanding of the molecular signaling pathways involved in PD, particularly within the context of how genetic and environmental factors contribute to the initiation and progression of this disease. The involvement of molecular chaperones, autophagy-lysosomal pathways, and proteasome systems in PD are also highlighted. In addition, emerging therapies, including pharmacological manipulations, surgical procedures, stem cell transplantation, gene therapy, as well as complementary, supportive and rehabilitation therapies to prevent or delay the progression of this complex disease are reviewed.

## Background

Parkinson disease (PD) is second to Alzheimer’s disease as the most common age-related complex, idiopathic neurological disorder [1]. It is characterized by tremor, bradykinesia and muscle rigidity along with impaired gait, and posture [2–4]. In addition, about half of the PD patients also exhibit frontostriatal-mediated executive dysfunction, including deficits in attention, speed of mental processing, verbal disturbances, impairment of working memory and impulsivity [5]. Dopaminergic neuronal loss in the substantia nigra pars compacta (SNpc), and depletion of dopamine (DA) levels in the striatum represent the hallmark pathology of PD [6]. Experimental evidence indicates that the prefrontal cortex (PFC), anterior cingulate gyrus, and/or frontostriatal pathways are also affected by PD [7].

Although the exact mechanism of dopaminergic neuronal loss in SNpc is not well understood. Mitochondrial damage, energy failure, oxidative stress, excitotoxicity, protein misfolding and their aggregation, impairment of protein clearance pathways, cell-autonomous mechanisms and “prion-like protein infection” may be involved in the onset and progression of PD [3, 8, 9]. Among them, protein misfolding and its subsequent accumulation in intracellular spaces has become a leading hypothesis for PD [10, 11]. The major misfolded amyloid protein inclusion observed in the intracellular spaces of SNpc neurons in PD is the Lewy bodies (LB) [3, 11, 12], which contain several misfolded amyloid proteins, including alpha-synuclein (SNCA), phosphorylated tau (p-tau), and amyloid beta protein (Aβ) [11, 13]. Several environmental toxins are associated with sporadic PD (SPD), which can be partially mimicked in experimental animal models of PD, such as the use of 1-methyl-4-phenyl-1,2,3,6-tetrahydropyridine (MPTP) and paraquat [14, 15]. Unlike SPD, familial cases are rare, and do not follow the prescribed symptoms of PD, which makes it more difficult to understand the pathogenesis of PD [10, 16].

Although several new treatments for PD have been developed [17], none of them effectively halt the progression of PD. The few symptomatic treatments currently available, are appropriate for only a limited number of patients. Moreover, side-effects, short-life span, and permeability issues are the major problems for use of these drugs against PD. Interestingly, recent developments in stem cell transplantation [18–20] and gene therapies [21, 22] have drawn special attention as alternative strategies for treating PD. For example, genetically engineered DA-neurons have shown promising results in mouse models of PD [20, 23]. Similarly, using lentiviral or recombinant adeno-associated viral vectors (rAAV), scientists are able to correct some of the dysfunctional metabolic pathways involved in PD [24]. Further, a very recent development of the gene editing technique, clustered regularly-interspaced short palindromic repeats-associated protein 9 (CRISPR-Cas9), may prove useful for treating PD [22]. Given the pressing need for the development of new, rational therapies for PD, the focus of this review is to provide basic conceptual information on the molecular mechanisms underlying PD, which may assist in the design of more effective drugs or other treatment strategies.

## Global scenario and risk factors for PD

Although the symptoms and therapies for PD were first mentioned in the “Indian Ayurveda” (5000 BC) and Chinese medical text, “Nei-Jing” (500 BC), it was James Parkinson, a British physician for whom the disease is named accurately described it as “the shaking palsy” in 1817. Epidemiological studies have revealed that PD is world-wide and affects 1–2% of those older than 65 years, and 4–5% of those aged over 85 years [8, 25]. In US, more than one million cases have been reported [26]. PD is more common in men (about 1.5 times) than in women [8], and a higher incidence of PD has been reported in developed countries [14], due to an increase in the aged population [14, 27]. Aging is the most dominant risk factor for PD. As such, the cases of PD are very low in people under 40 and becomes more prevalent in individuals in their 70s and 80s [10]. People with one or more close relatives who have PD have an increased risk of developing the disease themselves, but the total risk is still just 2–5%, unless the family has a known gene mutation for the disease [26]. Other risk factors exist, including exposure to environmental toxins [14, 27]. However, most scientists agree that PD is not, by itself, a fatal disease, but rather, it causes a worsening of normal functioning with time. Interestingly, the average life expectancy of a PD patient is generally the same as for normal people [28].

## Symptoms of PD

**Fig. 1:**
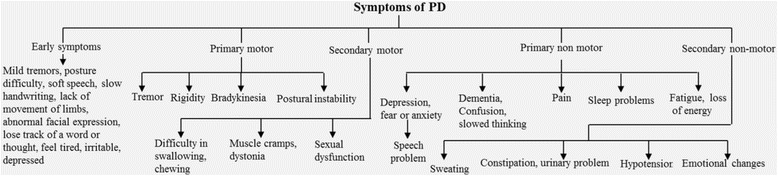
Different symptoms of PD. The PD symptoms are categorized into five major subtypes: early, primary motor, secondary motor, primary and secondary non-motor symptoms

The progression of symptoms in PD may take 15 to 20 years or more, but may vary person-to-person [8]. The major symptoms observed in PD patients are categorized into: (i) early symptoms; (ii) primary motor symptoms, (iii) secondary motor symptoms, (iv) primary non-motor symptoms, and (v) secondary non-motor symptoms (Fig. 1).

### Early symptoms

The early symptoms are subtle and progress slowly, making them difficult to detect. They include mild tremors, posture difficulty, soft speech, slow handwriting, lack of limb movement, abnormal facial expression, loss of focus in thought and speed, fatigue, irritability or depression, without provocation or cause [2, 29]. Sometimes the person may be stiff, unsteady, or unusually slow, and as the disease progress, the shaking or tremor may appear, which start from one side of the body, they eventually spread bilaterally over time [2, 29]. Generally, family members or close friends, or daily caretakers are more likely to detect the emergence of early symptoms in patients.

### Primary motor symptoms

(i) *Resting Tremor.* Shaking of hands, arms, legs, jaw, head, tongue, lips, chin are the primary motor symptoms observed in PD (Fig. 1). About 70% of people with PD experience “resting tremor” in the early stage of the disease, either in the hand or foot on one side of the body, or less commonly, in the jaw or face [26, 30]. As the disease progresses, both arms may become affected [26, 30]. Typically, the tremor takes the form of a rhythmic, back-and-forth motion at a rate of 4–6 Hz.
(ii) * Rigidity.* The rigidity or increase in stiffness or tonicity of a muscle is the second most common symptoms noted in PD patient [31]. The person with PD often feels stiff or weak, pain and cramping in muscles and joints. Sometimes muscle rigidity can cause an increase in resistance to the extent that the person feels as if someone else is moving his or her joints [31].
(iii) * Slow movement (bradykinesia).* Bradykinesia in PD causes unplanned movements, decreases in the extent of movement, or the slowing and loss of spontaneous and automatic movements [4]. Common bradykinesia includes a diminution of their handwriting (micrographia), decreased facial expression, decreased rate of eye blinking, and a soft or lowering of volume in their speech [4]. Sometimes it impairs simple tasks, such as routine movements [4]. Other symptoms include incomplete movement, difficulty initiating movements, and sudden stopping of ongoing movement [4, 29].
(iv) * Balance and coordination problems.* Impairment of coordination, including losing reflex mechanisms, causes instability or imbalance when the PD patient is standing [31]. In severe cases, PD patients are unable to get up off the ground after falling and have difficulties in making turns or abrupt movements [30].

### Secondary motor symptoms

Secondary motor symptoms include stooped posture, a tendency to lean forward, dystonia, fatigue, impaired fine and gross motor coordination, decreased arm swing, akathisia, cramping, drooling, difficulty with swallowing and chewing, and sexual dysfunction [8].

(i) *Difficulties in swallowing and chewing.* PD patients often have difficulties in swallowing due to losing control of muscle movement around the mouth and throat, which makes it difficult to chew solid foods. This prevents peristaltic movement of GI tract, thus constipation may develop in PD patient [32].
(ii) * Muscle cramps and dystonia.* A variety of pain, aches, muscle spasms or dystonia have been observed in PD [33]. These muscle cramps can be sustained for prolonged periods and can be very painful. Muscular rigidity is the principal reason for this, which may be exacerbated due to the side-effects of certain medications [34].
(iii) * Sexual dysfunction.* Sexual dysfunction is one of the major reasons for deterioration of quality of life of a PD patient. Hyper-sexuality, erectile dysfunction, and difficulties in ejaculation are found in some PD male patients. Whereas the loss of lubrication and involuntary urination during sex are common in female PD patients [35]. The tremor, bradykinesia, muscular rigidity, dyskinesia, hyper-salivation, and sweating may be the reasons for sexual dysfunction in PD [36]. In contrast, hyper-sexuality reported in male PD patients may be due to side-effects of medications [36].
(iv) * Changes of speech and voice.* About 90% of the PD patients have difficulties with voice control and are unable to deliver speech appropriately [37]. They may speak too softly or in a monotone, or may have slurred speech and develop a breathy or hoarse quality [32]. PD patients may hesitate before speaking, slur or repeat their words, or may even speak so fast that is difficult to understand them [32]. Communication difficulties are common during walking or doing any other tasks. Sometimes expression of complicated sentences become difficult for them, along with presence of longer pauses in their conversation [32, 37].

### Primary non-motor symptoms

Frequently observed non-motor symptoms in PD patients include depression, insomnia, and cognitive dysfunction.

(i) *Depression.* Depression is a common problem and an early indicator of PD, which can manifest itself before other symptoms appear [38, 39]. PD patients often experience episodes of sadness and depression, which results in an unpleasant attitude, without any apparent reason, which can reduce the quality of life. The level of depression can be sufficiently severe for some PD patients to have suicidal thoughts and ideations [38].
(ii) *Dementia and or cognitive dysfunction.* About half of the PD patients have cognitive dysfunction, slowness of thought processing [40]. During their conversation, PD patients have difficulties in finding the right words and in understanding complex sentences [40, 41]. Due to this “tip-of-the tongue” problem, PD patients often have many pauses during conversation, and their audience has a difficult time following their line of thought. This dementia may affect memory, social judgment, language, reasoning, or other mental skills [40, 41].
(iii) * Problems in sleep (insomnia).* Impairment of sleep is very common, and almost 80% of people with PD have difficulty staying asleep at night, or suffer from some form of restless sleep, nightmares, emotional dreams, drowsiness, or sudden sleep onset during the day [42, 43]. Muscle rigidity, tremors or stiffness at night, or frequent urge to urinate, or experiencing vivid dreams or hallucinations, including violent nightmares may underlie the interference in normal sleep for PD patients [29, 42, 43]. The most common sleep disorders include insomnia, REM sleep behavior disorder, sleep apnea, “sleep attacks, and restless legs syndrome” [43, 44].

### Secondary non-motor symptoms

(i) *Gesture and emotional changes.* Many people with PD have issues in reorganizing words and are unable to deliver their message or express their emotions appropriately [45]. Sometimes their facial expressions do not match the context of their speech or their voice intonation [46]. Furthermore, emotional breakdowns make PD patient fearful, insecure, and uncomfortable. Sometimes they are unable to cope with new environments prefer was not to travel, and to avoid socializing with friends. Also, many PD patients have developed personality problems, with their body gestures, their broken or “flattened voice”, and their disrupted emotional control, leading to misinterpretations about their capabilities, and sometime becoming targets for public ridicule [45, 46].
(ii) * Urinary problems and constipation.* PD patients often complain about dysfunction in urination and defecation [47]. Movement of smooth muscles in urinary bladder and gastrointestinal (GI) tract are often impaired, which can lead to urination problems and constipation. Constipation can occur because of the slow movement of gastrointestinal tract in PD patients [47].
(iii) * Sweating and skin problems.* Because of improper function of autonomic nervous system, the PD patient has difficulties in controlling body temperature, which sometimes causes excessive sweating [48]. The face of PD patients become very oily, particularly on the forehead and at the sides of the nose. Sometimes the scalp can become excessively oily, as well, resulting in dandruff, and in some cases, the skin become very dry, rough, and wrinkled [48].
(iv) * Blood pressure.* PD patients also suffer from increased incidences of cardiovascular diseases [49]. For example, when a PD patient stands up from a lying-down position, his or her blood pressure decreases suddenly, causing dizziness, lightheadedness, and, in extreme cases, loss of balance or fainting [49]. The effects of some medications can be another reason for the sudden dropping of blood pressure [49].
(v) *Pain.* PD patients often complain of pain in muscles and joints, which may be due to muscle rigidity and abnormal postures [50]. Treatment with dopaminergic agonists can cause aggravate the pain in muscles and joints, along with unexplained burning and stabbing sensations [51].

## Causes of motor impairment in PD

**Fig. 2:**
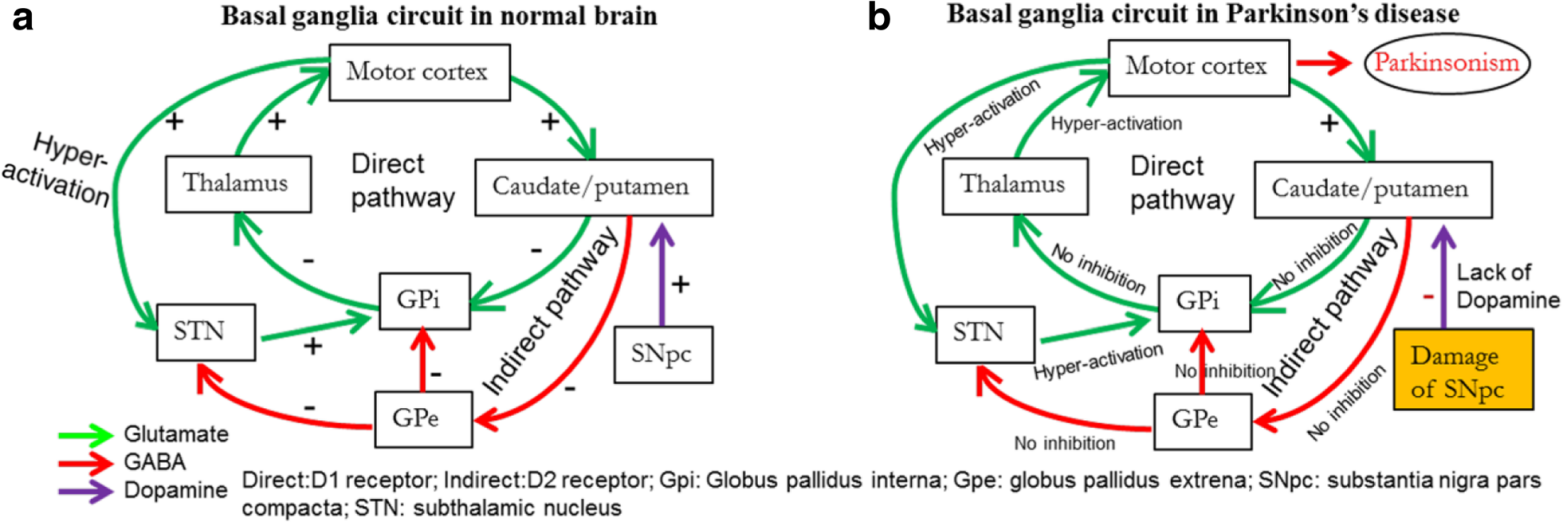
Neuronal circuits and neurotransmission mechanisms of control in the brains of normal individuals and those with Parkinson’s disease. **a**: Neuronal circuit in basal ganglia in normal brain. **b**: Degeneration of substantia nigra pars compacta (SNpc) impairs cortico-striatal circuit in PD brain. Decrease in DA levels in the SNpc and striatum causes loss of control of striatal neuronal firing, leading to withdrawal of inhibitory effects on globus pallidus as well as thalamus, therefore, the thalamus becomes over-excitable, which activates the motor cortex excessively. This ultimately leads to impairment of motor coordination and causes Parkinsonism

(i) *Role of dopamine.* The principal brain area affected by PD is the substantia nigra, pars compacta (SNpc), a vital part of the basal ganglia [52]. This area is predominantly composed of neurons which secrete DA, an essential brain monoamine, which functions primarily as an inhibitory neurotransmitter. In healthy brain, DA regulates the excitability of striatal neurons, which are involved in controlling the balance of body movement. In PD, DA-neurons of SNpc degenerate, and DA levels are diminished [52, 53]. Inadequate DA levels cause less inhibition of the activity of striatal neurons, allowing them to fire excessively. This makes it difficult for PD patients to control their movements, leading to tremor, rigidity, and bradykinesia, the hallmarks of PD-associated motor symptoms [3] (Fig. 2).
(ii) * Role of serotonin.* Other than DA, serotonin (5-HT) also plays an important role in PD development, especially in several motor and non-motor symptoms, including tremor, cognition, depression, and psychosis, as well as L-DOPA-induced dyskinesia [54]. A reduction of 5-HT levels in the PFC has been observed up to 18 weeks following an acute injection of MPTP in mice [55]. Similarly, a decline in 5-HT transporter (SERT) levels has been reported in the cortex and anterior cingulate following unilateral striatal lesions in the macaque monkey [56]. In addition, a reduction of SERT-immunoreactive axons in the PFC reduced 5-HT-imunoreactivity in median raphe neurons, or reduced PFC SERT binding capacity have also been observed in brains of PD patients [57, 58]. Furthermore, there is ~25% loss of serotonergic receptor (HT1A) at median raphe nucleus in PD patients, and this is correlated with the severity of resting tremor [59], which suggests that 5-HT projections in midbrain is more relevant for initiation of PD tremor than loss of nigrostriatal DA-projections. Recently, we have shown that 5-HT turnover in the PFC may play a pivotal role in executive dysfunction in MPTP-model of PD [60]. Similarly, a strong relation between decline of 5-HT and depression have been found by several investigators in PD [61], however, the importance of 5-HT and it relationship with the progression of PD warrants further attention.
(iii) * Role of acetylcholine.* Acetylcholine (ACh), which plays significant role in cognition, is downregulated in several neurological diseases, including PD and AD [62]. Within the basal forebrain subventicular region, there is a broad band of cell clusters, commonly known as nucleus basalis of Meynert (nbM), which are predominantly cholinergic in nature. Different patterns of neuronal loss have been observed in the nbM of patients with PD, LBD, AD, or other forms of dementia, which strongly supports the idea of an involvement of the cholinergic system in PD [62, 63]. Importantly, the presence of LB and neuronal loss were found the nbM of postmortem brain tissue of PD patients with cognitive decline, which suggests that the cholinergic system is also involved in the cognitive dysfunction observed in PD [61].
(iv) * Role of GABA/Ca2* ^*+*^ *system.* The gamma amino butyric acid (GABA) is an inhibitory neurotransmitter, which controls the calcium (Ca^++^) influx directly via GABAergic receptors and, indirectly, via astrocytes network [64]. The Ca^++^/GABA mechanism stabilizes neuronal activity both at the cellular and systemic levels. In case of PD, due to mitochondrial damage, Ca^++^-buffering system become impair, which causes Ca^++^-excitotoxicity leading to neuronal loss in the SNpc [65], whereas the Ca^++^-buffering is controlled by GABA activity [66]. It has been observed that ~80% of newly diagnosed PD patients have abnormal olfaction, which is due to damage of the DA-neurons in the olfactory bulbs [67]. The function of the DA-neurons both, in the midbrain and in the olfactory system are controlled by glial cell-derived neurotrophic factor (GDNF), which is also regulated by the Ca^++^/GABA system. Moreover, GDNF function as a chemo-attractant for GABAergic cells and a strong chemo-attractant for axons of DA. The neuroprotective effects of GDNF was observed in PD animal models when administered in GABAergic neurons in the striatum, but not in the SNpc [68], suggesting collapsing of GABA/Ca^++^ system are involved in DA-neuronal death in PD [69].

## Molecular mechanisms of PD

**Fig. 3:**
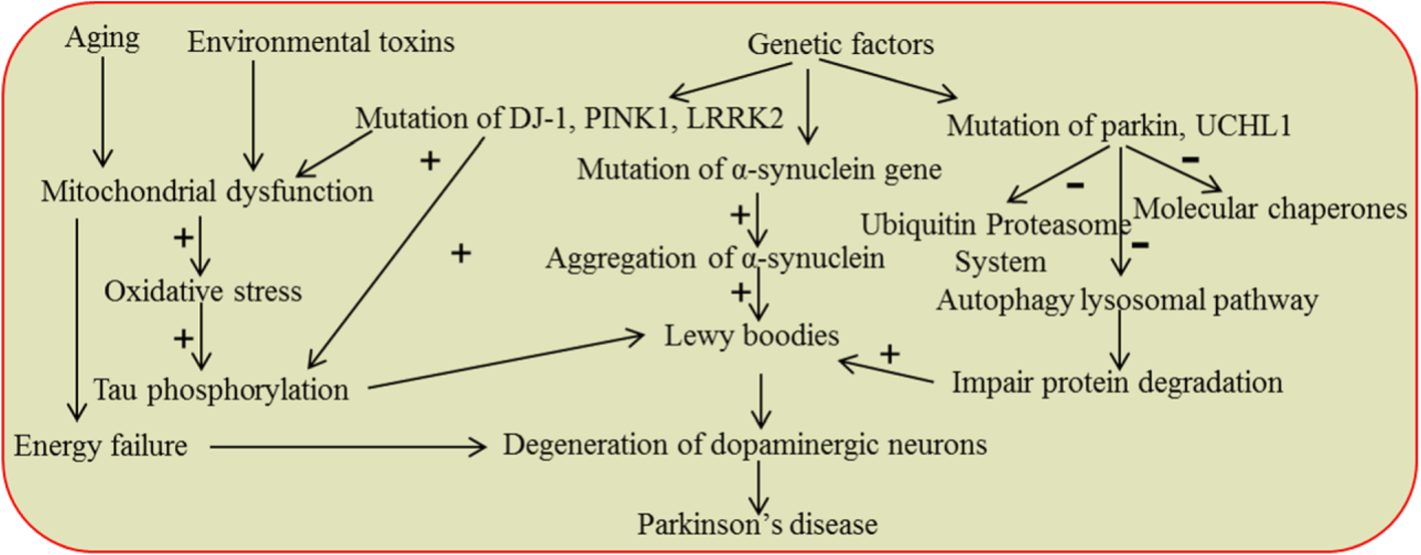
Schematic diagram showing the involvement of different factors and signaling pathways for degeneration of DA-neurons in PD

PD is a multifactorial disease (Fig. 3), where both genetic and non-genetic, such as environmental factors, are involved [16, 25, 27]. The most salient mechanisms involved in the development of PD include the accumulation of misfolded proteins aggregates, failure of protein clearance pathways, mitochondrial damage, oxidative stress, excitotoxicity, neuroinflammation, and genetic mutations [6, 13, 70].

### The role of aggregation of misfolded proteins in PD

**Fig. 4:**
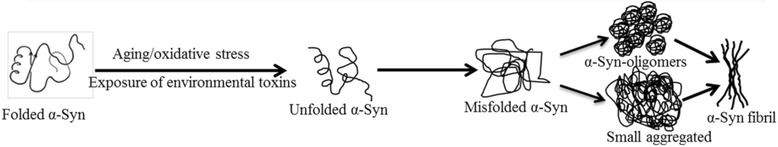
Schematic diagram showing the steps that cause an accumulation of SNCA. Natural SNCA becomes misfolded under stress and is deposited as oligomers, small aggregates, or fibrils, which play a significant role in DA-neuronal loss in PD

(i) *Aggregation of alpha-synuclein (SNCA).* One of the hallmark pathologies of PD is the intracellular accumulation of LB in DA neurons of the SNpc [70], which contain misfolded aggregates of SNCA and other associated proteins [13]. Interestingly, several molecular, genetic and biochemical studies evidenced that a mixture of multiple misfolded protein aggregates, such as p-tau, Aβ, and SNCA, are frequently seen in human post-mortem brains of patients who were neuropathologically diagnosed as having mixed dementia with Lewy bodies (DLB) and PD with dementia (PDD) [67]. Gomperts and colleagues examined the brains of several PD patients and found a mixture of amyloid deposition in their brain, which was linked to cognitive declines without dementia, suggesting amyloid contributes to cognitive, but not motor decline over time [68]. Similarly, Hepp and colleagues found that the load and extent of Aβ pathology contribute to cognitive impairments in PDD and LBD [69].
  The oligomers, proto-fibrils, and fibrils of SNCA or other misfolded amyloid proteins can make a pore in the membrane, causing neuronal death via oxidative stress, energy failure, excitotoxicity, and neuroinflammation [13, 71] (Fig. 4 **)**. For example, a single intracerebroventricular (i.c.v.) infusion of SNCA oligomers (α-SYOs) in mice causes the development of late motor and non-motor symptoms, such as deficits in the pole and rotarod tests, along with reduced TH and DA content in the caudate putamen [72]. Similarly, mutations of SNCA gene (e.g. A53T, A30P, E46K and H50Q) cause familial PD with its early onset, rapid progression, and a high association of dementia [73]. Overexpression of SNCA in animal and cell culture models showed an accumulation of SNCA aggregates in mitochondria, marked deficits in mitochondrial motility, and decreased mitochondrial membrane potential [74]. The SNCA knockout mice showed mitochondrial lipid abnormalities and impairment of electron transport chain [75], and the mice became less sensitive to mitochondrial toxins [76]. Furthermore, A53T, a transgenic mouse model of PD develops neuronal mitochondria degeneration with accumulation of SNCA-containing mitochondria and marked reduction of complex IV activity [77]. Mitochondrial DNA damage, respiratory chain dysfunction, oxidative stress, along with SNCA inclusion have also been reported to observe in human DA-neurons in the PD brain [78].
(ii) *Tau.* Hyper-phosphorylation of tau (p-tau) can cause an accumulation of paired helical filaments of tau, known as neurofibrillary tangles (NFT), a hallmark pathology of different neurodegenerative diseases, including AD, frontotemporal dementia with parkinsonism (FTDP), and progressive supra-nuclear palsy (PSP) [79]. The FTDP is linked to chromosome 17 (FTDP-17), with p-tau accumulation occurring in cortex and SNpc areas [79]. The p-tau can also be co-localized with LB, which is often associated with the development of sporadic PD [80]. Similarly, in the case of FTDP, a mutation of gene coding for microtubule associated protein (MAPT) causes an increase in the accumulation of p-tau [80]. The p-tau also has been linked to the LRRK2 gene mutations [16]. Although NFTs are associated most closely with AD, they can co-localize with SNCA in LB and play an important role in destabilization of DA-neuronal architecture, which ultimately leads to rapid degeneration and death of DA neurons [79, 81, 82].

### Role of gene mutations in PD

A plethora of recent studies, including the discovery of gene mutations in familial or inherited forms of PD, demonstrate that 5–10% of late-onset forms of PD are linked to genetic factors [16, 24] (Table 1). The most common PD-related genes are SNCA, parkin, DJ-1, PINK1 [16]. Experimental and clinical evidence suggest that there are five different chromosomes (5, 6, 8, 9, and 17), which are linked to an increase in susceptibility to develop PD. For example, the *parkin* gene is located on chromosome 6, which contains genes that are associated with early-onset of PD [16]. Further, some of the PD patients who do not respond to L-DOPA treatment have specific genes located on chromosome 9 [26]. Similarly, the late-onset PD is related to chromosome 17 (FTDP-17), adjacent to the gene for tau [83]. In addition, gene encoding ubiquitin carboxyl-terminal hydroxylase (UCH-L1), and genes on chromosomes X, 1, 2, and 4, also have influential roles in the etiology of PD in some families [16].

(i) *Parkin.* Parkin is an important protein associated with protein clearance pathways, such as ubiquitin-proteasome system, which can help degrade misfolded proteins in the cell (Table 1). Parkin acts as an E3 ubiquitin ligase, which can bind covalently with ubiquitin on various misfolded protein substrates to aid in their degradation [84]. Parkin also has been co-localized with SNCA, and can form LB inclusions [85]. In contrast, parkin mutations can cause aggregation of misfolded amyloid proteins within SNpc [85]. Parkin-deficient mice and parkin mutations in idiopathic PD patients show loss of neurons in the locus coeruleus of the midbrain, [85]. Furthermore, in the case of autosomal recessive juvenile Parkinsonism, parkin mutations can cause a significant decline of ubiquitin-ligase enzymatic activity in the SNpc [86, 87], which can decrease the proteasomal degradation process significantly. In addition, parkin is also involved in the regulation of the release of DA from SNpc [6].
(ii) * DJ-1 (PARK7).* DJ-1, a dimer consists of 189 amino acids, is localized in the cytoplasm, nucleus, and mitochondria, and has been linked to early-onset of PD [88] (Table 1). It is neuroprotective, including regulation of activity of certain cell survival-related genes (PI3 K/Akt pathway), transcriptional regulation, anti-oxidant, chaperone and protease activity, as evidenced by several in vitro studies [89]. DJ-1-deficient mice show locomotor deficits, decreased activity in the D2 type of DA receptors, and enhances sensitivity to MPTP [68]. Similarly, DJ-1 deletions and point mutations cause development of autosomal recessive PD [15]. In addition, DJ-1 has been co-localized with SNCA, p-tau, indicating DJ-1, which may play a key role in synucleinopathies and tauopathies [69]. Furthermore, DJ-1 can bind to several chaperones, including HSP70, carboxy-terminus of HSP70-interacting protein (CHIP), and mitochondrial HSP70/mortalin/Grp75, and can help in the degradation of misfolded SNCA [70]. DJ-1 also modulates the expression of the human TH gene by sequestering the transcriptional repressor, poly-pyrimidine tract-binding protein-associated splicing factor (PSF) from the human TH gene promoter, in order to maintain TH levels in DA neurons of SNpc [88].
(iii) * PINK1 (PARK6).* PTEN-induced putative kinase-1 (PINK1), is a 63 kDa serine/threonine-protein kinase, which is localized in the mitochondria and protect neurons from stress-induced mitochondrial damage [90]. In vitro studies suggest that PINK1 can act as a cell-survival factor. The PINK1 gene mutation has been observed in several families with PD, in which it causes an increase in cell vulnerability [90, 91]; (Table 1). Mutations of PINK1 gene are also linked to mitochondrial dysfunctions and degeneration of SNpc neurons, which ultimately leads to the development of PD [90].
(iv) *LRRK2/PARK8* (dardarin)*.* Leucine-rich repeat kinase 2 (LRRK2), is a 268 kDa multi-domain protein, which is encoded by the *PARK8* gene. Several point mutations on the PARK8 gene have been linked with late-onset of PD [16] (Table 1). *Post-mortem* tissue from PD patients show several point mutations in PARK8, with significant DA neurodegeneration, with or without the presence of LB aggregation. In addition, the p-tau pathology observed in *post-mortem* brains of PD patients may be linked to mutations of the LRRK2 gene [16].
(v) *PARK3, PARK9, PARK10, and PARK11.* Familial PD is also related to mutation of the PARK 3-, 9-, 10- and 11- genes (Table 1). For example, the onset of late-stage SPD is linked with a mutation of the PARK3 gene. A few cases have linked PARK9 mutation with PD in one Jordanian family, whereas, PARK10 has been linked to PD in Icelandic families [16] (Table 1).
(vi) *Glucocerebrosidase (GBA) gene mutation. GBA* is considered one of the most common genetic risk factors associated with Parkinsonism. Recently, Velayati and colleagues reported that the mutations of GBA gene are associated with not only the development of PD, but also for LBD [92]. The GBA mutations are associated with alterations in lipid levels, leading to lysosomal storage disease, which can induce synucleinopathies, and also autophagy-lysosomal dysfunction [92]. Similarly, we also found a mutation of GM1 synthase or the upregulation of ganglioside-3 synthase (GD3S) are associated with decreases in the neuroprotective ganglioside (GM1) and increases in toxic gangliosides (GD3 and GT3 series), which can induce neurodegeneration in SNpc in MPTP-lesion mice [93].
(vii) *Mutation of mitochondrial DNA (mtDNA).* The mitochondria is a target organelle in PD, and an increase in age-related mtDNA mutations has been observed in PD brain tissue [94]. A group of researchers have developed mitochondrial gene-replacement therapy to replace the mutated human mitochondrial genes as a potential treatment for PD and other sporadic neurodegenerative diseases [95]. This form of therapy could slow down the progression of the type of PD that is closely related to mitochondrial dysfunction.
(viii) *Other gene mutations:* Mutations of autophagy-related genes, which encode vacuolar protein sorting protein-associated protein 35 (VPS35), are also associated with a rare form of autosomal dominant PD [96]. Similarly, several other genes, including TMEM, COMT, IF4G1E, GRIN2A, GSTP1, TNF-α, COX-2, SLC6A3, ADH1C, rs356219, SREBF1 and SREBF2, HLA-DRB5, BST1, GAK, ACMSD, STK39, MCCC1, SYT1, CCDC62/HIP1R are also involved in development of PD.

**Table 1:** Genetic causes of Parkinson’s disease

Gene	PARK loci	Chromosome	Form of PD	Mutations and their origin	Refs.
SNCA	PARK 1	4q21	Autosomal dominant	A30P (Germany), E46K (Spain), A53T (Greece, Italia, Sweden, Australia, Korea), A18T (Poland), A29S (Poland), E46K (Spain) H50Q (UK), G51D (France)	[197, 234–238]
Parkin	PARK 2	6q25.2–q27	Autosomal recessive, juvenile	Various mutations, exonic deletions, dupli/triplications (Japan)	[239, 240]
Unknown	PARK 3	2p13	Autosomal dominant	Europe	[241]
SNCA	PARK 4	4q21	Autosomal dominant	Duplication and triplication USA	[242]
UCHL1	PARK 5	4p14	Autosomal dominant, idiopathic	I93M and S18Y (Germany)	[192, 234, 243]
PINK1	PARK 6	1p35–p36	Autosomal recessive	G309D, exonic deletions (Italy)	[244]
DJ-1	PARK 7	1p36	Autosomal recessive, early onset	Homozygous exon, deletion L166P (Europe)	[245–248]
LRRK2	PARK 8	12q12	Autosomal dominant, idiopathic	R1441C ⁄ G ⁄ H, Y1699C G2019S, I2020T, G2385R (Japan)	[249–251]
ATP13A2	PARK 9	1p36	Kufor–Rakeb syndrome, early onset	Loss-of-function mutations (Jordan, Italy, Brazil)	[252–254]
Unknown	PARK 10	1p32	Idiopathic	(Iceland)	[255]
Unknown	PARK 11	2q36–q37	Autosomal dominant, idiopathic	(USA)	[256]
Unknown	PARK 12	X	Familial	(USA)	[257]
HTRA2	PARK 13	2p13	Idiopathic	A141S, G399S (Germany)	[258, 259]
MAPT	MAPT	17q21.31	Familial	79 of Ser/Thr of tau (tauopathies) (Asian, USA)	[238, 260]
Glucocerebrosidase-1	GBA-1	1q21	Recessive	Lysosomal storage disorders (USA)	[238, 92, 261]
Other genes	TMEM, IF4G1E, GRIN2A, GSTP1, TNF-alfa, COX-2, SLC6A3, ADH1C rs356219, SREBF1 and SREBF2, COMT HLA-DRB5, BST1, GAK, ACMSD, STK39, MCCC1, SYT1, CCDC62/HIP1R [262]				

### PD caused by impairment of protein degradation pathways

**Fig. 5:**
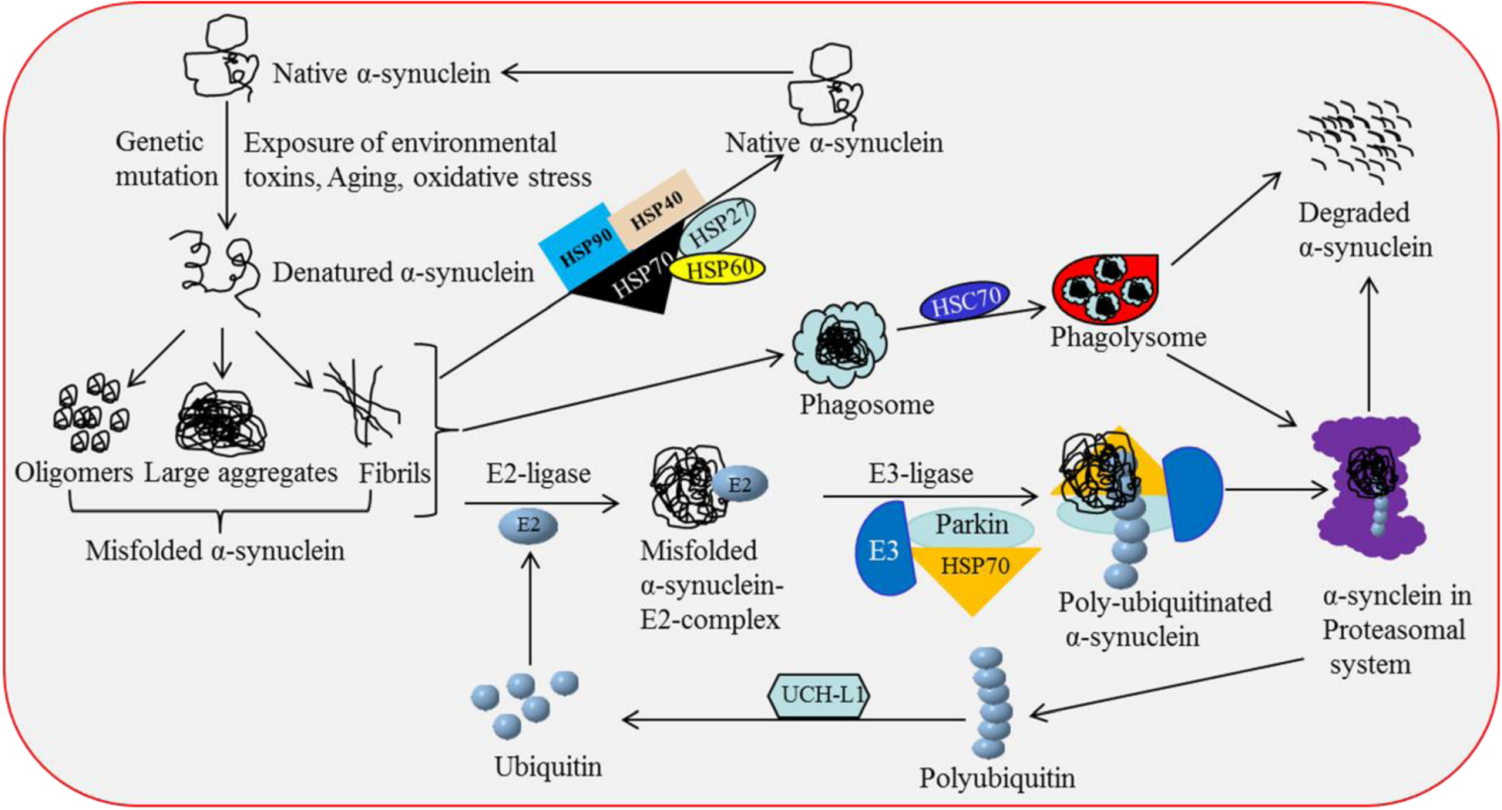
Role of protein clearance pathways in PD. Different protein clearance pathways, including molecular chaperones (HSPs), ALP (including macro-autophagy, micro-autophagy and chaperone-mediated autophagy), and the ubiquitin-proteasomal system in degradation of misfolded proteins, such as SNCA and LB have been associated with PD

**Fig. 6:**
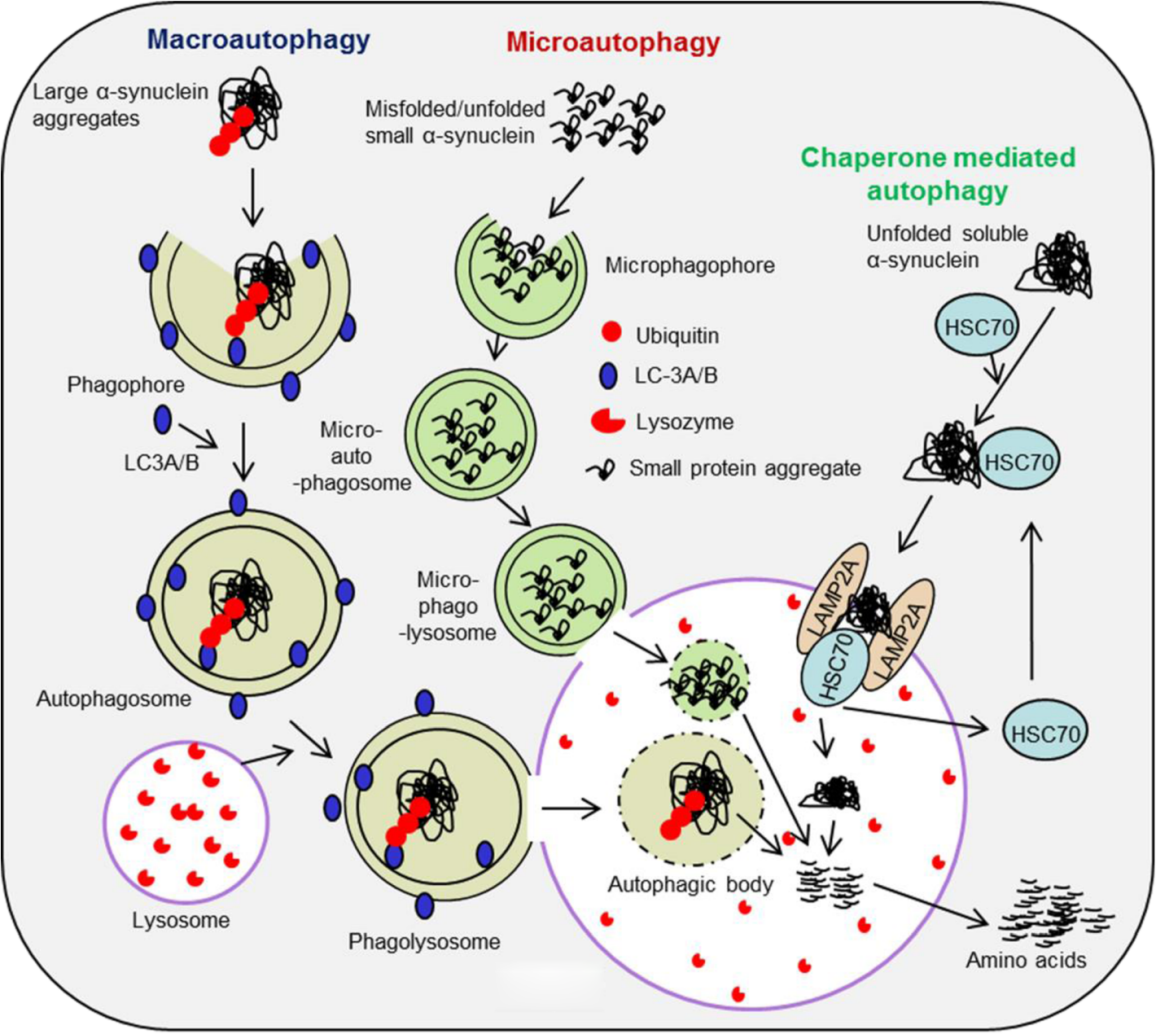
Role of autophagy-lysosomal pathway in degradation of misfolded protein aggregates in PD. Insoluble, larger and smaller SNCA/LB aggregates are degraded by macro-autophagy and micro-autophagy, respectively, whereas soluble, small misfolded SNCA and or LB are degraded by CMA

(i) *Ubiquitin-Proteasome System (UPS).* UPS is the most efficient disposal system of cell, and is mainly responsible for degradation of short polypeptides into small intracellular and plasma-membrane proteins in normal cells [97]. It is also responsible for degradation of misfolded or damaged proteins in the cytosol, nucleus, or endoplasmic reticulum [98]. Impairment or failure of this critical cellular system has been observed in the pathogenesis of PD, leading to aggregation of misfolded amyloid proteins, such as LB, and an increase in neurodegeneration in the SNpc [99, 100]. In the case of PD, several other proteins, such as parkin and UCH-L1, along with UPS, are involved in the degradation of misfolded SNCA. Experimental evidence suggests that UCH-L1 is involved in the production of ubiquitin, which has been co-localized with LB [101] (Fig. 5). Role of UPS in LB degradation in PD can be best studied by inhibiting the UPS system.
  For example, the inhibition of proteasome system by using lactacystin resulted in a deposition of LB and degeneration of DA neurons in the fetal rat ventral mesencephalic cells [102, 103]. A retrograde patients of DA neurodegeneration has also been observed in rodent brains, following intrastriatal administration of lactacystin [104]. Similarly, inactivation of ubiquitin hydrolases with ubiquitin aldehyde produce toxic effects in primary neuronal cultures [97, 102]. Furthermore, low levels of proteasome inhibition (100 nM MG115) in human neuroblastoma cells (SH-SY5Y) for several weeks showed mitochondrial degeneration, elevated levels of protein oxidation and aggregates [105, 106], resembling sporadic PD, which strongly supports the role of UPS dysfunction in PD pathogenesis. Furthermore, subcutaneous injections of either the naturally occurring proteasome inhibitor epoxomicin (1.5 mg/kg) or the synthetic proteasome inhibitor PSI (peptidyl aldehyde, selective inhibitor of the chymotrypsin-like activity of the proteasome, 3 or 6 mg/kg) over a period of 2 weeks in adult Sprague-Dawley rats induces progressive motor dysfunction, along with loss of DA nerve terminals in the striatum and a progressive reduction of the DA transporter ligand [107]. To prove the link between the UPS and PD, researchers also developed genetic models of PD, such as *parkin-*mutated mice, although this mouse model lacks overt signs of parkinsonism [108]. Similarly, inactivation of UCHL-1 in mice did not produce DA neurodegeneration, but did result in axonal dystrophy syndrome or motor ataxia [109]. Interestingly, *parkin* mutations in Drosophila exhibit selective DA-neuronal death, as well as locomotion deficits, mimicking those of PD patient [110, 111].
(ii) *Molecular chaperones (Heat shock proteins, HSP).* The molecular chaperone, is one of the most efficient, highly conserved cellular defense mechanisms involved in protein folding, refolding of partially misfolded proteins, and protein degradation [112, 113]. Major HSPs involved in PD are HSP 26, 40, 60, 70, 90 and 100. Some of the HSPs are localized in synapses and axons, and their levels are down-regulated in PD [114] as well as other neurodegenerative diseases [113]. Importantly, HSPs can bind to aggregated SNCA or tau oligomers or pre-fibrillar structures, and interfere by forming low MW soluble oligomers or higher order insoluble structures [115, 116] which reduce their toxicity (Table 2). HSPs also play pivotal roles in the regulation and precise functioning of ubiquitin proteasome and the autophagy-lysosomal pathways [113, 116, 117].
  In drosophila and yeast models of PD, HSP70 co-expression prevents DA cell death by decreasing the SNCA toxicity [118], whereas mutations of ATPase domain in HSP70 (K71S) increase toxicity [119]. Similarly, over-expression of HSP70 decreases MPTP- or rotenone-induced neurotoxicity in rat brain slices [120] and also in cultured SK-N-SH or PC12 cells [121]. Furthermore, a reduction of total and detergent-insoluble fractions of misfolded SNCA aggregates were observed in an in vitro model of PD, which co-express different yeast HSPs (HSP104, HSP40, HSP27, or HSP70) [122], suggesting molecular chaperones become dysregulated in PD.
(iii) *Autophagy lysosomal pathway (ALP).* Because they are too large to pass through the narrow proteasome barrel, large protein debris, such oligomers and fibrils of SNCA, cannot be degraded through UPS [123, 124]. Autophagy, which includes macro-, micro-, and chaperone-mediated autophagy (CMA), are the specialized mechanisms serve as alternative protein clearance machineries present in every cell for degrading LBs in PD [123, 125, 126] **(**Fig. 6 **)**. Micro-autophagy is mainly involved in degradation of small cytosolic proteins, even under resting conditions, and macro-autophagy is responsible for degradation of large aggregates. CMA is more specific, performing its activity by interacting with heat-shock cognate protein (HSC70), which specifically bind to small soluble proteins to be degraded via specific pentapeptide targeting motif (KFERQ). The HSC70 docks the t0-be-degraded proteins to the lysosomal membrane receptor, lysosome-associated membrane protein 2 (LAMP2A), and then transport them into the lysosomes, where they are degraded by lysozymes [127]. Experimental evidence suggests that the down-regulation of autophagy-related genes, Atg5 or Atg7, in the CNS leads to aggregation of poly-ubiquitinated protein debris in neurodegenerated tissue in mice [128, 129].
  It has been shown that the SNCA is selectively translocated into the lysosomes for degradation by the CMA [123]. Therefore, dysfunction of CMA decreases the efficiency of SNCA degradation, causing excess accumulation of this protein, which impairs neuronal activity significantly. Further, PD brain is particularly vulnerable to dysfunction of autophagy-lysosomal pathway (ALP), which may be due to the failure of autophagosome formation or its inability to bind with lysosomes, due to deficiency of lysozymes, or dysfunction of HSC70 or LAMP2A [6, 130, 131]. Substantial evidence from human *post-mortem* studies reveals that autophagy mechanisms become impaired in PD brain. For example, accumulation of autophagy vacuoles [132] and levels of the ALP markers, microtubule-associated protein 1 light chain 3 (LC3) [133] have been reported to increase in the SNpc area of *postmortem* PD brain and temporal cortex of patients with DLB [134] in comparison to age-matched controls, suggesting dysfunction of autophagy is linked with PD progression. Furthermore, decreased levels of LAMP1, LAMP2A, and HSC70 have been observed in the SN of PD patients, suggesting CMA dysregulation [135, 136]. Recent transcriptome studies with *postmortem* tissue have revealed that several autophagy-related downstream mechanisms, such as mTOR and PI3K/AKT signaling, were also severely affected in PD brain [137, 138].

**Table 2:** Different molecular chaperones (HSPs), localization, functions and their involvements in PD

HSPs	MW (kDa)	Location in cell	Co-localization	Functions	Refs.
HSP27	20–30	Cytosol, ER, nucleus	SNCA, Tau	Protein degradation	[263, 264, 116, 265]
HSP40	40	Cytosol,	SNCA	Protein folding	[116, 263]
HSP60	60	Mitochondria	SNCA	Prevent protein aggregation	[116, 263, 266]
HSP70	70	Mitochondria, Cytosol, ER, nucleus	SNCA	Protein folding and unfolding	[116, 118, 263, 265, 267, 268]
HSP90	90	Cytosol, ER,	SNCA	Protein degradation and transcription factor	[116, 263, 265, 269]
HSP100/104	100–110	Cytosol, ER,	SNCA	Thermal tolerance	[116, 263, 270]

### Role of environmental toxins on PD

Recent yield-boosting advances in the agricultural and fertilizer industries have led farmers to use various types of the pesticides, sometimes indiscriminately, for their crop production. Exposure of those environmental toxins (herbicides, pesticides, fungicides, insecticides etc.) has contributed to the development of SPD [14, 27] (Table 3). Importantly, farmers and people living in rural areas are vulnerable to PD, due to exposure of those toxins, either through direct contact or through drinking water. Many people are also exposed to bacterial toxins, viruses or illegal street drugs, such as the synthetic heroin (MPTP or 1-methyl-4-phenyl-1, 2, 3, 6-tetrahydropyridine), leading to SPD [5, 128].

**Table 3:** Different environmental toxins involved in neurodegeneration and Parkinsonism

Toxins	Use	Mode of action	Effects on nervous system	Refs.
MPTP	Herbicide	Inhibit electron transport	Parkinsonism	[140, 153, 271]
Rotenone	Pesticide, insecticide	Interfere with Mitochondrial electron transport system	Parkinson’s like symptoms	[143, 272]
Paraquat	Herbicide	Interfere electron transport, photo synthesis	Oxidative stress	[15, 273, 274]
Maneb	Fungicide	Interferes glucocorticoid metabolism	Parkinson’s like symptoms	[274–276]
Zineb	Pesticide	Metabolized to carbon disulfide-a neurotoxin	Convulsions, tiredness, dizziness weakness, headache, fatigue, slurred speech, unconsciousness	[277–279]
Ziram	Pesticide	Unknown	Prolonged inhalation causes neural and visual disturbances	[141, 279, 280]
Thiram	Pesticide	Unknown	Convulsions, headaches, dizziness, fatigue drowsiness, confusion	[279–281]
Nabam	Fungicide	Unknown	Convulsion, dizziness, confusion	[279, 282]

**Fig. 7:**
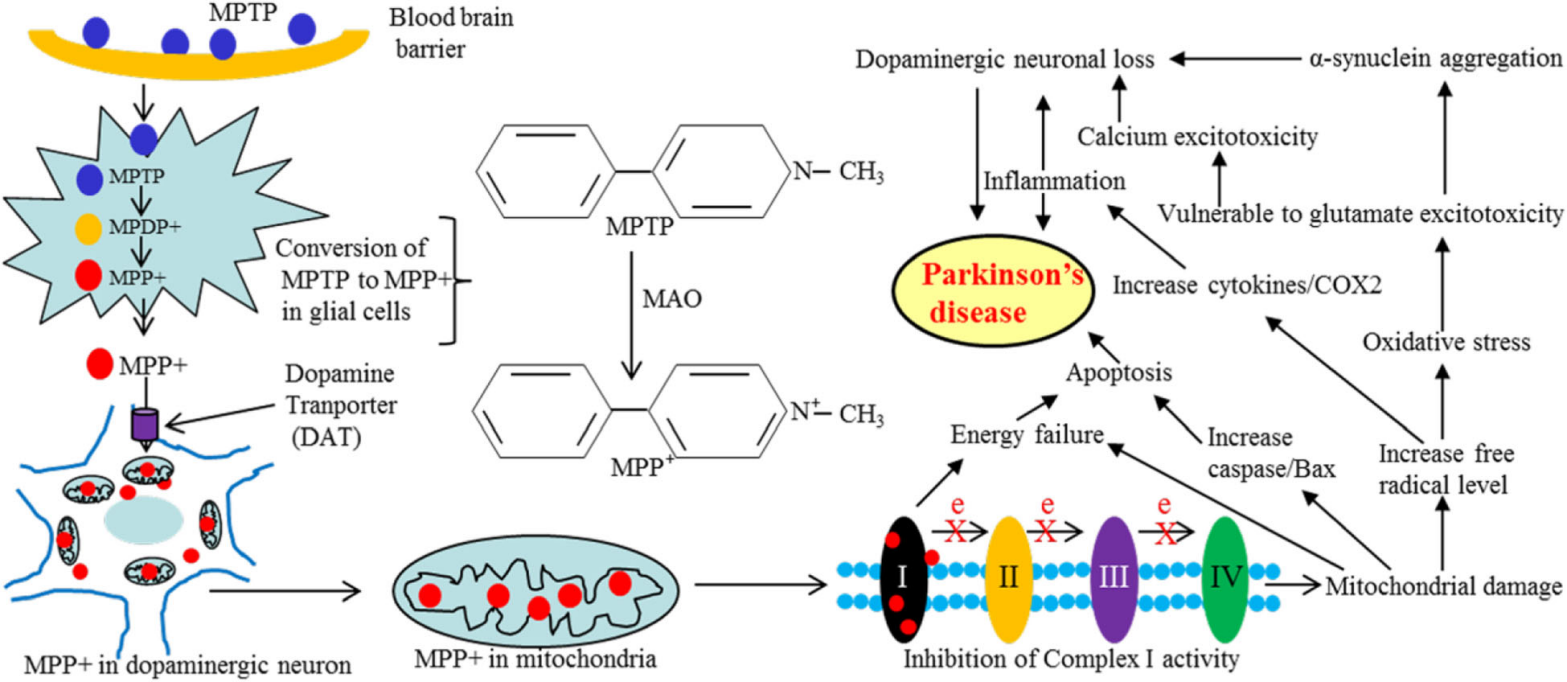
Mechanistic details of MPTP-induced DA-neuronal loss in PD. After crossing blood brain barrier, MPTP enters glial cells, where it is converted to MPP+. This MPP+ then enters neurons and damage mitochondria, which causes energy failure, oxidative stress, glutamate and Ca^++^ excitotoxicty, increased aggregation of misfolded SNCA, and DA-neuronal loss

Exposure of MPTP into the cell produces MPP^+^, the actual toxic metabolite, which can pass through DAT and thus, attack DA-neurons in SNpc and induce parkinsonism [139]; (Fig. 7). Because of this capability, currently MPTP is widely used to produce a severe, permanent parkinsonian syndromes in animal models of PD [140]. There are structural similarities between MPTP and different environmental toxins, such as paraquat, maneb, zineb, nabam, thiram, ziram, and rotenone (Table 3), and many of these environmental toxins may produce Parkinsonism in animals [14, 141].

**Fig. 8:**
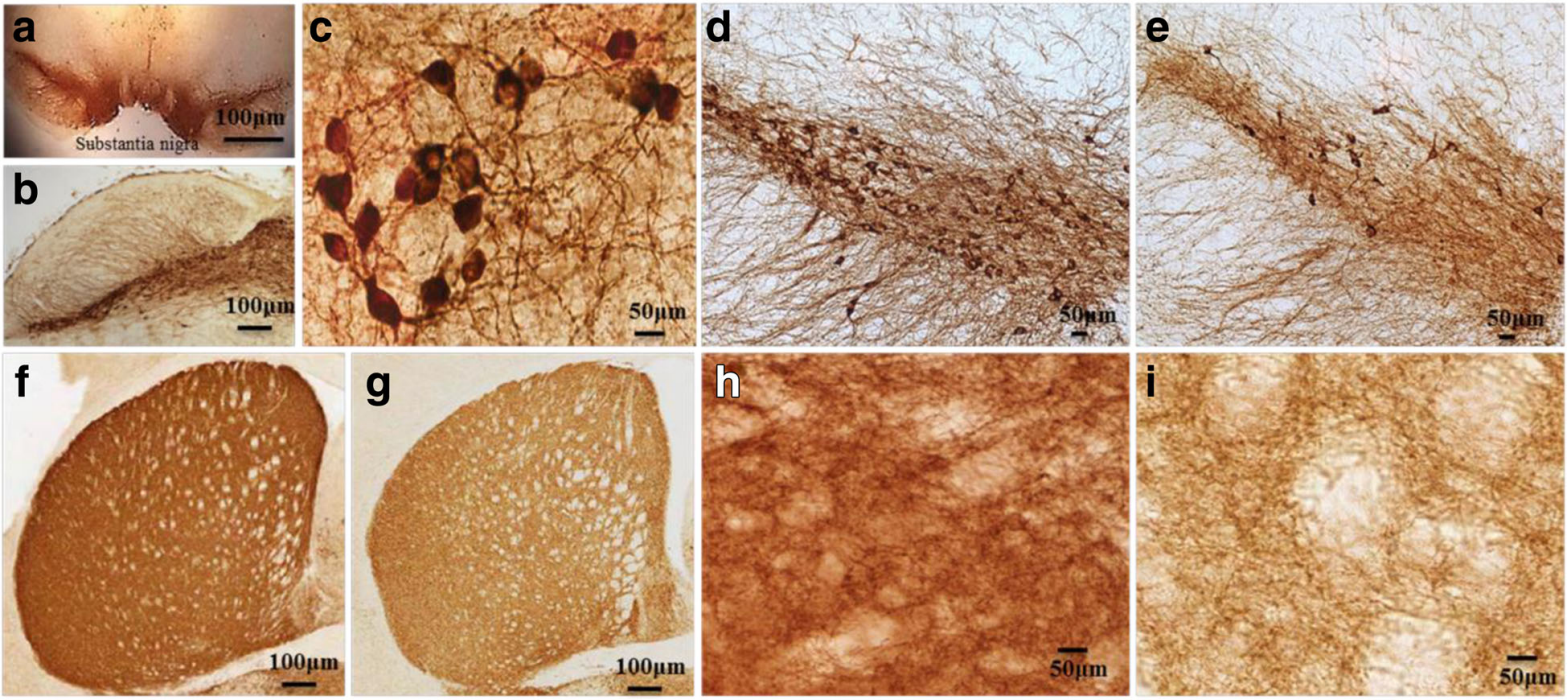
Brain areas affected by PD. Substantia nigra in mouse brain (**a** and **b**); TH+ DA-neurons in SN (**c**; 40 x); in control (**d**) and MPTP-treated mouse brain (**e**). TH+ fibers in control (**f**, **h**) and MPTP-treated (**g**, **i**) mouse striatum. Note: The loss of DA-neurons in SN (**e**), along with loss of TH+ fibers in striatum, have been observed after MPTP treatment (**g** & **i**)

For example, in one of our experiments, mice that were injected with MPTP (25 mg/kg BW) for 5 days, showed an 80% loss of TH-immunoreactivity in SNpc 5 weeks later, indicating a substantial loss of DA-neurons in that area. We also observed TH-positive DA-fibers were more sparse in the striatum (Fig. 8), which suggests that, due to loss of DA neurons in SNPc, the DA fibers become diminished in the striatum [3]. Similarly, injecting 6-hydroxydopamine (6-OHDA), into the striatum can also produce PD-like symptoms in rodents [142]. Similarly, rotenone is another well-known component of pesticides that causes degeneration of DA neurons in SNpc, due to energy failure much like what happens with MPTP exposure [143]. Several studies have shown that the most environmental toxins can inhibit the complex-I activity and interfere the mitochondrial electron transport system, which can ultimately increase free radical production, leading to oxidative stress [140].

**Fig. 9:**
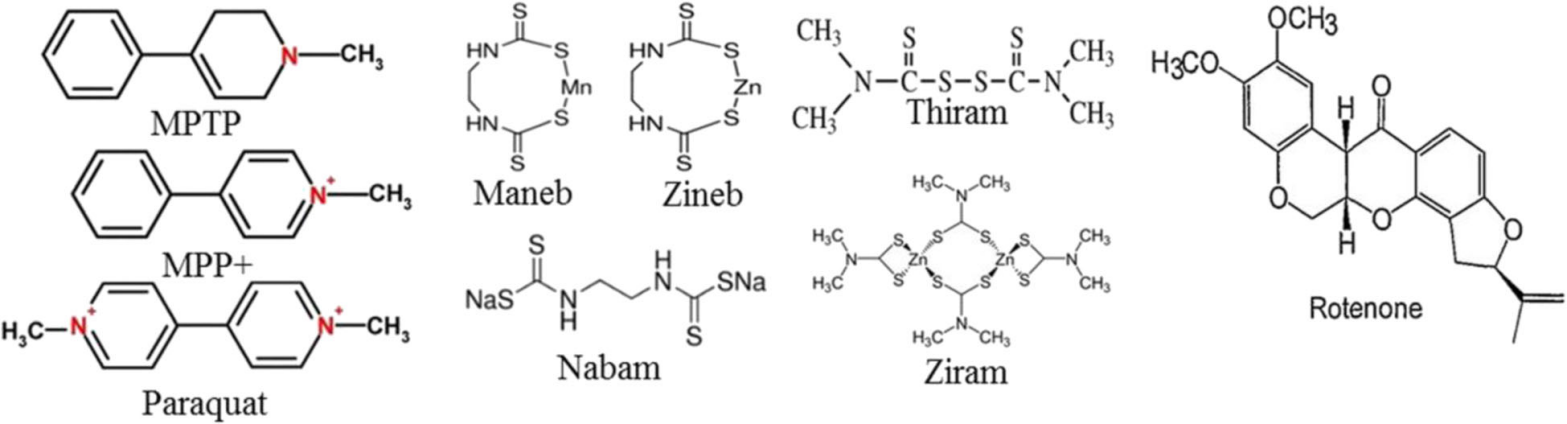
Different ETs-associated with PD. Chemical structure of different pesticides, herbicides, fungicides, and insecticides which may produce Parkinson-like symptoms in animal models

Although several investigators have developed many animal models of PD using environmental toxins, none of them demonstrated the primary PD symptoms, like resting tremor or bradykinesia. They also fail to accurately recapitulate the mechanisms of DA neuronal death (Table 4) (Fig. 9).

**Table 4:** Different animal models and their applications in PD research. LB-Lewy bodies, IFC-impaired fear conditioning, CD-cognitive deficits, MD-mitochondrial, deficits, RA-reduced anxiety, ASP-affected synaptic plasticity, RD-reduced dopamine level

Category	Models	Mechanism	NS loss	Inclusions	Motor deficit	Non-motor deficit	Applications		Demerits		Refs.
Environ mental toxins	6-OHDA	Complex I inhibition	+++	_	+++	Cognitive, psychiatric, and GI disorders	Screen therapies for PD, study mechanisms of cell death		Degeneration non-progressive		[283]
	MPTP	Complex I inhibition	++	-, presence of SNCA at SNpc	+++	Numerous	Screen therapies for PD, study mechanisms of cell death		Non-progressive rare inclusions		[284, 285]
	Rotenone	Complex I inhibition, ↑ROS	++	Presence of SNCA at SNpc	+++	Decrease GI motility	Test neuroprotective compounds		morbidity, mortality, time consuming & laborious		[286]
	Paraquat	Complex I inhibition, ↑ROS	+++	No inclusions at SNpc	_	Not detected	Test neuroprotective compounds		Substantial morbidity, mortality, time consuming & laborious		[287, 288]
	Maneb	Impairment of glutamate and DA uptake	+	_	+	Not detected	Study glutamate uptake in DA neurons		No inclusion, less DA neuronal damage		[287, 288]
Others	SHH, Nurr1, Pitx3, EN1	Impaired protein synthesis in DA-neurons	++	_	+/−	Not known	Study the mechanism of Translation in DA neurons		No SNCA		[289–291]
	MitoPark	Mitochondrial deficit	++	+/−	+	Not known	Study the role of mitochondria in PD		Less motor deficit		[292, 293]
	PDC	EAATs blockade, excitotoxicity, ↑ROS	++	_	+	Not known	To study excitotoxicity and Oxidative pathway in PD		No SNCA		[293, 294]
Genetic	Parkin (PARK2)	Loss of ubiquitin E3- ligase activity	+/−	+/−	+/−	_	Study the role of E3 ligase in PD		No inclusion, less DA neuronal damage		[239, 295]
	LRRK2 (PARK 8)	Loss of enzymatic activity	_	_	Drosophila +	Not detected	Study the role of LRRK2 mutations related to PD		No SNCA nor no DA degeneration		[296, 297]
	PINK (PARK6)	Mitochondrial damage	+/−	+/−	+/−	Not detected	Study the role of mitochondria in PD		No SNCA or no DA degeneration		[298, 299]
	DJ-1(PARK 7)	Increase ROS, Mito. dysfunction	+/−	+/−	+/−	Not detected	Study oxidative stress & mitochondrial dysfunction in PD		Less inclusion & DA neuronal damage		[300, 301]
SNAC mutation and animal models of PD											
Models	Promoter		Background			SNCA	Motor signs	Nonmotor signs	TH neurons loss	Disease progression	Ref
WT, A53T	PDGF-b		C57BL/6 9 DBA2			+	+	–	+	–	[302–304]
A53T	Mouse Thy-1		C57BL/6			LB	+	–	–	–	[305]
WT, A30P, A53T	Mouse Thy-1		C57BL/6			+	+	+	–	+	[306, 307]
WT, (A30P)	Mouse Thy-1		C57BL/6 x DBA2			+	+	IFC	+	+	[303, 308]
Y39C	Mouse Thy-1		FVB/N			+	+	CD	–	+	[309]
A30P + A53T	Human Thy-1		C57BL/6 x DBA2			+	+	–	+	+	[310]
(WT), (A30P), A53T	Mouse prion		C3H/HeJ 9 C57BL/6 J backcrossed into C57BL/6 J PARKIN KO			+	+	MD	–	+	[311, 312]
WT, A53T	Mouse prion		C57BL/6 x C3H			+	+	RA	–	+	[313, 314]
(WT), A53T	Mouse prion		FVB/N, FVB 9129, SNCA-KO			–	+	–	–	+	[315]
(WT), A30P	Mouse prion		C57BL/6 J 9 DBA2 backcrossed into C57BL/6 J			–	+	ASP	–	–	[316]
WT, A30P, A53T	Hamster prion		C57BL/6 J x SJL			–	+	–	–	+	[317, 318]
WT, A30P, A53T	Rat THP		Swiss Webster x C57BL/DBA			–	–	–	–	–	[287]
WT, A30P ± A53T	Rat THP		C57BL/6			–	+	–	–	+	[319]
WT, A30P, A53T	CaM-tTA (tet-off)		C57BL/6 (WT and A30P), C57BL/CH3 (WT and A53T)			–	+	CD	–	+	[320, 321]
A30P	KI in endogenous SNCA		C57BL/6			–	+	–	RD	+	[322]
WT, A30P, A53T	Endogenous SNCA (BAC)		FVB/N 9129S6 / SvEvTac			–	+	+	–	+	[323]

Moreover, these toxins can cause acute or rapid cell death, unlike progressive neurodegeneration noted in PD [14]. In addition, most therapeutics used to protect against the neurodegeneration caused by these environmental toxins in animal models are unable to translate into effective human therapies. Therefore, the selection of a toxin to produce an animal model of PD is a challenging task (Table 4).

### Role of mitochondrial damage and oxidative stress in PD

**Fig. 10:**
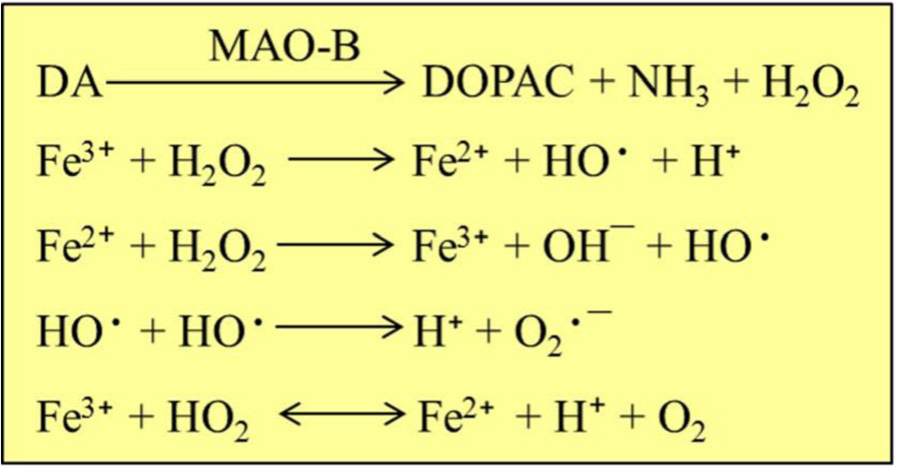
Oxidative stress theory in PD. With the help of MAO-B, the DA is converted to DOPAC and produces hydrogen peroxide (H_2_O_2_). The H_2_O_2_ is then converted to other ROS by Fenton reaction

One of the most promising theories in PD research, as well as other age-related neurodegenerative diseases, is the oxidative stress theory [144]. This theory posits that the mitochondria is the “hot-spot” for degenerative processes. In PD, the abnormal activity of complex-I in mitochondria has been observed, which directly interferes with cellular ATP production, leading to cell death [145]. In addition, the brain monoamines, such as DA and 5-HT, generally act as antioxidants [146]. However, breakdown of DA by monoamine oxidase-B (MAO-B), and combined with ground state O_2,_ leads to the formation of ROS [147] **(**Fig. 10 **)**. Researchers have found increased oxidative stress markers and related changes (including free radical damage to DNA, proteins, and fats) in PD patients [147]. In addition, increased levels of the apoptotic marker protein, “Bax” has been observed in DA-neurons of the SNpc in MPTP-treated mice [94]. Recently, investigators have developed hybrid cells, called “cybrid”, to check the role of mitochondria in development of PD [148]. They have placed mitochondrial DNA from PD patients into neuroblastoma cells and found these cybrids develop LB, just like those in the DA-neurons of PD patients. Similarly, certain gene mutations that are involved in cell-survival mechanisms may lead to impairment of mitochondrial activity and ATP production. These findings provide strong support for the idea that mitochondrial defects play a key role in the development of sporadic PD.

### Role of excitotoxicity in PD

DA is an inhibitory neurotransmitter, which normally maintains the excitation status of the subthalamic nuclei (STN) at basal levels. However, in the case of PD, due to deficiency of DA neurons, the STN become over activated, leading to excessive production of neurotransmitter glutamate [149]. Excessive glutamate binds to its ionotropic receptors (NMDA or AMPA) and open the voltage-gated calcium (Ca^++^) channels (VGCC), which causes Ca^++^ excitotoxicity. Excess Ca^++^ load can damage the mitochondria and produce ROS, leading to oxidative stress [150, 151]. In addition, environmental toxins, can cause increased production of glutamate, leading to Ca^++^ excitotoxicity which makes DA neurons vulnerable to neurodegeneration [14, 94, 141, 151].

### Neuroinflammation involved in PD

**Fig. 11:**
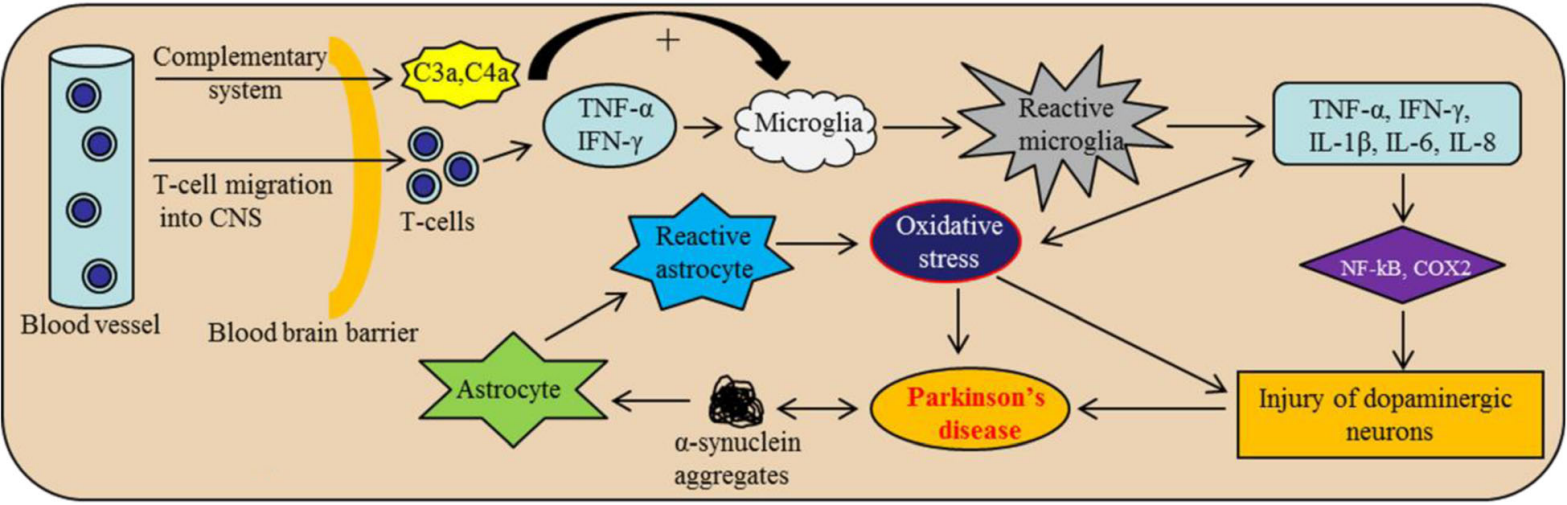
Mechanism of neuroinflammation in PD. T-lymphocytes and complementary systems can activate microglia to secrete several cytokines, which causes DA-neuronal injury. Similarly, aggregated SNCA can also activate astrocytes, which causes oxidative stress, leading to neuronal injury

A cascade of events are involved in neuroinflammation processes in PD, including activation of microglia and an increase secretion of cytokines [152]. For example, researchers have found strong links between pro-inflammatory cytokines and degeneration of DA neurons, following sub-chronic administration of MPTP in animals [153]. Several clinical studies have shown that the level of inflammatory enzymes, such as cyclo-oxygenase-2 (COX-2), is increased several times in DA-neurons of the *postmortem* PD brain and in a mouse models of PD [154] (Fig. 11 **)**.

#### Prion hypothesis

During the past few decades, scientists have postulated several mechanisms for the onset and progression of PD. Recently, the “prion hypothesis” is considered one of the most intriguing theories behind its onset. This theory posits that SNCA spreads throughout the CNS, similar to “prion proteins” and infect adjacent new, healthy neurons and that this cycle continues until most of the CNS neurons are infected. Therefore, “prion-like infection” of SNCA may be responsible for the progression and neurodegeneration of some types of PD [11]. According to Braak’s hypothesis, SNCA, bacteria, or viruses can travel via the olfactory tract and into the Vegas nerve to the medala and spread throughout the CNS, which can responsible initiate sporadic PD [155]. Recently, Chandra and colleagues discovered that enteroendocrine cells (EECs) in gastro-intestinal track, possess many neuron-like properties and that SNCA is expressed in the EEC lines, as well as in native EECs of mouse and human intestines. These cells directly connect to SNCA-containing nerves, forming a neural circuit between the gut and the nervous system (gut-brain interaction hypothesis) [156]. Moreover, abundant clinical and pathological evidence have localized misfolded SNCA in EECs, before it appears in the brain. These phenomena suggest that PD pathogenesis may originate in the gut and spread to the CNS via cell-to-cell “prion-like propagation” [156]. Although the “prion hypothesis” provides useful insights into the progression of PD, the presence of SNCA is not always necessary for the emergence of PD pathology or parkinsonism. Therefore, even though SNCA can infect healthy cells like a prion, the “prion hypothesis” of PD remains controversial [157].

## Diagnosis of PD

Most clinicians face difficulties in diagnosing PD accurately, because some of the symptoms can emerge during normal aging. Moreover, only a few tests are available, which can help to diagnose PD. Therefore, most PD patients are diagnosed on the basis of their medical history and certain neurological examinations [2]. The presence of LB is one of the most definitive ways to diagnose PD [158], which can be done by microscopic examination of *postmortem* brain tissues. However, LB can also be found in brains of patients who lack other symptoms without parkinsonism [159]. For example, more than 8% people over 50 years, 13% of people over 70, and 16% of over 80 years of age show LB in their brains, in the absence of any other symptoms of PD [160]. Therefore, the presence of LB in the brain tissue is not the sole indicator of PD, but an accurate diagnosis requires the presence of at least two of the three major motor signs, such as resting tremor, rigidity, and bradykinesia [161] (Table 5). The examination of different reflexes and limb movements are also considered as standard tests for the diagnosis of PD [161]. These can be measured by different means: (i) bradykinesia can be tested by measuring the capability to clamp finger and thumb together, or tap foot up and down; (ii) tremor index can be determined by simple inspection; (iii) muscular rigidity can be tested by moving the neck, upper limbs, and lower limbs; (iv) postural instability can be tested by “pull test”. Overall, the progression of PD can be diagnosed and categorized by different stages, as described by Hoehn and Yahr scale [162] (Table 5).

**Table 5:** Different stages of development of symptoms in PD as described by Hoehn and Yahr

Stages	Characteristics
Stage-I	Signs and symptoms on one side only; tremor of the limb; minute changes in posture, locomotion, and facial expression.
Stage-II	Symptoms are both sides; minimal disability; posture and gait affected
Stage-III	Slowing of body movements; early impairment of equilibrium on walking or sliding; generalized dysfunctions.
Stage-IV	Severe symptoms; can still walk to a limited extent; rigidity and bradykinesia; unable to live alone; tremor may be less than in earlier stage.
Stage-V	Cachectic stage; invalidism complete; cannot stand or walk; requires constant nursing care.

## Treatment of PD

Like other neurodegenerative diseases, PD can cause socioeconomic and emotional breakdown to the immediate family members, caretakers and friends of the patient. Unfortunately, effective therapeutics are not currently available, but early diagnosis and appropriate palliative treatment can provide for a more productive and longer life for most PD patients. Currently, several therapies are available to slow down the disease progression, albeit modestly, or provide transient relief of the severe symptoms of PD. Physicians most commonly use either (i) drug treatment or (ii) surgery to treat PD, however, some accessory therapies are also available.

### Drug treatments in PD

**Fig. 12:**
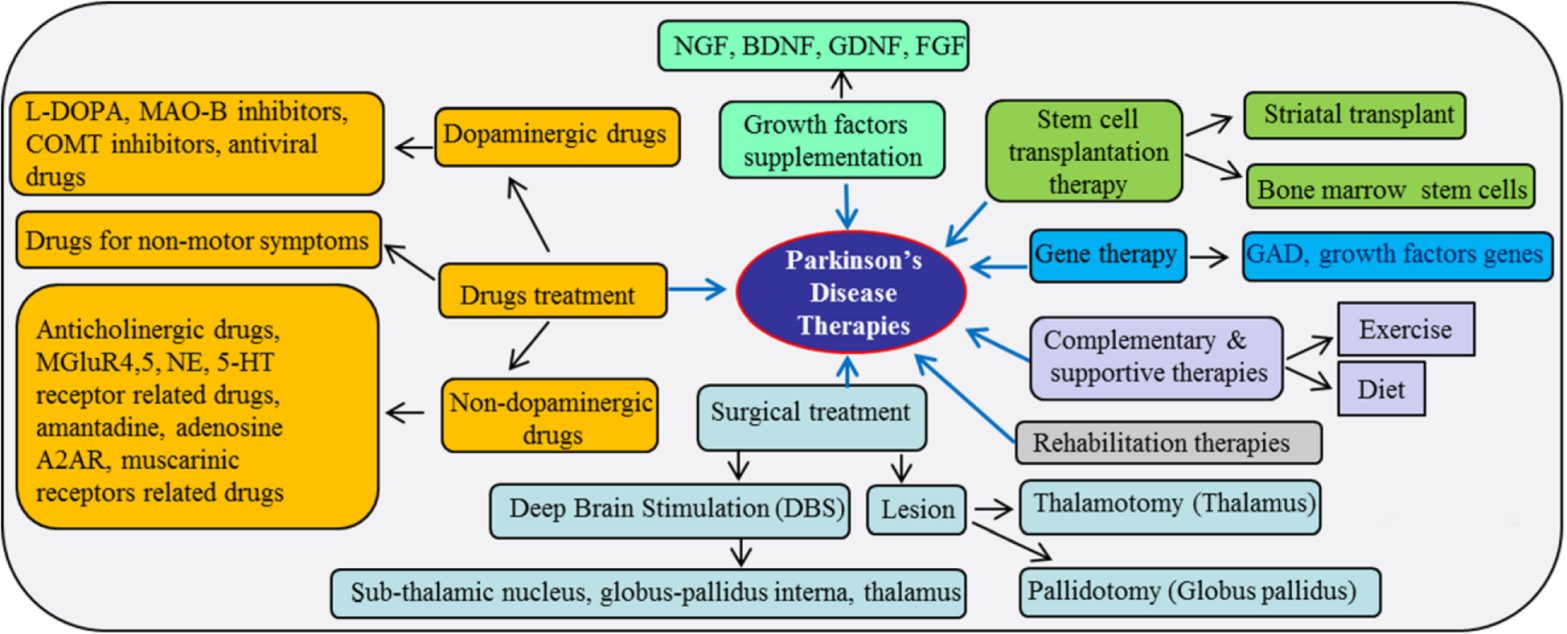
Possible therapies for PD. Currently different therapies available for treating PD include pharmacological manipulations, surgical treatments, stem cell and gene therapies, rehabilitation therapies and other complementary and supportive therapies

Although several generic drugs are available to reduce PD pathogenesis, the most critical factor is the selection of right dose. Therefore, during recommended course of treatment, a physician always assesses the effect of drug on the daily life of the patient, because drugs may take time to affect the patient’s body. Recently microinjection or infusion techniques allow several neuro-active substances to be injected into the damaged brain areas of PD [163]. Currently, the available medications for PD are divided into two categories: (1) dopaminergic drugs; (2) non-dopaminergic drugs (Fig. 12).

#### Dopaminergic drugs

**Fig. 13:**
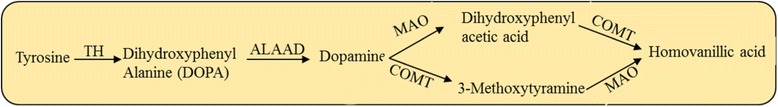
DA-biosynthesis and degradation. TH: Tyrosine hydroxylase, ALAAD: Aromatic L-amino acid decarboxylase, MAO: Mono amine oxidase, COMT: Catechol O-methyl transferase

(i) *Levodopa (L-DOPA).* Physicians usually prescribe DA-drugs to the PD patients in an effect to restore DA levels. As DA, itself, cannot cross the blood brain barrier, therefore, DA precursors, such as levodopa (L-3, 4-dihydroxyphenylalanine/L-DOPA) are commonly prescribed [164]. L-DOPA is very effective in reducing the “resting-tremors” and other primary symptoms [165], but L-DOPA is unable to preserve or replace degenerated DA-neurons or to stop further progression of PD [166]. Furthermore, it may cause nausea, vomiting, low blood pressure, restlessness, drowsiness or sudden onset of sleep (Table 6). In addition, because the conversion of L-DOPA to DA occurs very fast, decreasing its potency when it reaches the target area, physicians often prescribe carbidopa, which prolongs the therapeutic effect when given in conjugation with L-DOPA [167].
(ii) *MAO-B inhibitors.* Decrease DA levels in PD may be due to its rapid breakdown by the catalytic enzyme monoamine oxidase-B (MAO-B), whose level is increased in the PD brain [17] (Fig. 12). Therefore, inhibition of MAO-B is a good strategy to maintain DA levels in PD brain [168]. Selegiline, (L-deprenyl) and rasagiline are well-tolerated and the most commonly used MAO-B inhibitors [169], which, when administered with L-DOPA, and can sustain the response of L-DOPA even up to a year or more. Although several side effects have been reported with the use of these drugs (Table 5), they have promising effects on restoration of cell functioning or slowing down the loss of DA-neurons in PD [169].
(iii) *COMT inhibitors.* MAO can breakdown the DA to dihydroxy phenyl acetate, which is further catalyzed by the enzyme, catechol-O-methyl transferase (COMT), to form homovanillic acid (Fig. 13).
(iv) As COMT is responsible for breakdown of DA indirectly, therefore, inhibition of COMT could be another way to restore DA and treat PD [170]. The common COMT inhibitors are entacapone and tolcapone, which prolong the effects of L-DOPA by preventing the breakdown of DA [171]. These drugs also can reduce the sensitivity of L-DOPA in PD patient and produce fewer side-effects (Table 5) [171].
(v) *Dopamine agonists.* These drugs can increase DA levels in the brain and are most effective during the early stages of PD. They can also be combined with L-DOPA in late-stages of PD to increase the life span of L-DOPA [172]. The common DA agonists used to treat PD patients are pramipexole and ropinirole which are generally less effective than L-DOPA for controlling rigidity and bradykinesia [173]. Unfortunately, these drugs have several potential side effects that are similar to L-DOPA (Table 6) [172, 173].

**Table 6:** Some common drugs used in PD therapy, their mode of actions and effects and disadvantages

Drugs	Mode of action	Effects	Adverse side effects	Refs.
L-DOPA	Dopamine agonist	Increases dopamine concentrations	Nausea, vomiting, low blood pressure, restlessness, drowsiness.	[324, 325]
Selegiline	MAO-B inhibitor	Maintains L-DOPA levels	Dizziness, dry mouth, insomnia, muscle pain, rash, nausea, constipation, severe headache, tachycardia, arrhythmia, hallucinations, chorea, or difficulty in breathing.	[326]
Creatine	Boosts mitochondrial function	Antioxidant, prevents MPTP-induced neuronal damage	Nausea, stomach pain, diarrhea, muscle cramps; difficult breathing; swelling of face, lips, tongue, or throat, and weight gain.	[327]
Bromocriptine, Apomorphine, Pramipexole, Rropinirole	Dopamine agonist	Increases dopamine levels	Drowsiness, nausea, vomiting, dry mouth, dizziness, leg swelling, and feeling faint upon standing, drop in blood pressure, confusion, hallucinations, or psychosis.	[328]
Entacapone and tolcapone	preventing the breakdown of dopamine	prolongs the effects of L-DOPA	Hepatotoxic, nausea, diarrhea, orthostatic hypotension, urine discoloration and dizziness, mitochondrial dysfunction,	[329]
Amantidine	Activate dopamine synthesis	Increases dopamine levels	Blurred vision, confusion, difficult urination, dizziness, fainting seeing, and hearing, swelling of the hands, feet, or lower legs.	[330]
Rofecoxib	Inhibit COX-2	Prevents inflammation	Back pain, diarrhea, dizziness, headache, heartburn, and loss of energy or weakness, nausea, stuffy or runny nose, swelling of legs and feet, blurred vision, constipation.	[331]
ACP-103 (Pimavanserin)	Blocks serotonin receptors	Decrease levodopa-associated complications	Hyperprolactinemia, menstrual and sexual dysfunction, akathisia, distressful motor disturbance, restlessness	[332]
GM1 gangliosides	Dimerization of tyrosine kinase A and increases neurotropic factors	Increases dopamine	Not known	[333]
Quetiapine	Blocking of the dopamine type 2 (D2) and serotonin type 2 (5-HT2) receptors	Reduce psychosis and/or agitation	Agitation, dizziness, tremor, anxiety, hypertonia, abnormal dreams, dyskinesia, involuntary movements, confusion, amnesia, hyperkinesia, increased libido, abnormal gait, myoclonus, apathy, ataxia, hemiplegia, aphasia, buccoglossal syndrome	[334]
Ubiquinone or coenzyme Q10	Improves mitochondrial function	Antioxidant, slows disease progression in early-stages	Lower blood pressure, hemorrhage, skin itching, nausea, vomiting, headache or migraines, abnormal breathing, back pain, bronchitis, changes in attention, chest pain, constipation, coughing, diarrhea, dizziness, fainting, falling, fatigue, hearing loss, heart attack, indigestion, insomnia, irritability, loss of appetite, low energy, muscle pain, night sweats, respiratory tract infection, sore throat, urinary infection etc.	[335, 336]
S-Adenosyl-methionine (SAM)	Methylates phospholipid and increase nerve-cell communication	Improves dopamine transmission, decreases depression	Gastrointestinal disorders, dyspepsia, and anxiety.	[337]
Entacapone, tolcapone	COMT inhibitors	Inhibit DA breakdown	Diarrhea, nausea, sleeps disturbances, dizziness, urine discoloration, abdominal pain, low blood pressure, hallucinations.	[338]

#### Non-dopaminergic drugs

Non-dopaminergic drugs include anti-cholinergic compounds, norepinephrine (NE), serotonergic receptor- and muscarinic-receptor-related compounds, and antiviral drugs.

(i) *Anticholinergic drugs.* One of most important excitatory neurotransmitters in the brain is ACh, which has been reported to decrease in several areas of the brain parts in PD patients. In PD, DA levels are diminished, causing less inhibitory activity in the brain, allowing ACh-induced excitation to continue to the point of over-excitation. Therefore, anticholinergic drugs may be effective [174]. Although anti-ACh drugs can help to reduce tremors and muscle stiffness in PD, only about 50% of the patients get any relief, and this is only for brief periods, whereas only 30% of patients showing any symptomatic improvements [175]. Moreover, the anti-ACh drugs have several side effects (Table 6) [175].

#### *Other drugs*

The non-motor symptoms, including depression and anxiety, can be treated with anti-depressants. Benzodiazepine is one of the most commonly used drugs to treat anxiety in PD patients [176], but it has some side effects. Similarly, clozapine is prescribed to control dyskinesia in PD, but this can cause agranulocytosis and other side effects [177].

#### Immunotherapies

There are mainly two immunotherapeutic strategies available: active and passive, which have tested in animal models and human PD patients for targeting SNCA. Masliah and colleagues used a transgenic mouse overexpressing human wild type SNCA. These mice exhibit SNCA accumulation in neurons and glia of the neocortex, hippocampus, and SNpc [178]. Mice that were immunized with recombinant human SNCA showed a decreased in accumulation of SNCA inclusions in temporal cortex, and preserved synaptophysin-positive nerve terminals, as well as reduced neurodegeneration [179, 180] (Table 7).

**Table 7:** Alfa-synuclein immunization studies in animal models of PD Tg-trangenic, hSNCA-human alfa synuclein, rh-SNCA-recombinant human alfa synuclein, rAAV-recombinant adeno-associated virus, SN-substantia niagara, PDGF-platelet derived growth factor, TH-tyrosine hydroxylase, Ag-antigen, Ab-antibody, APOE-Apolipoprotein E

	Active immunization		Refs.
Animal models	Ag/Ab	Outcomes	
Tg-mice expressing hSNCA under the PDGF-β promoter, D-line	rh-SNCA	Reduction of accumulated SNCA in neurons and higher number of synaptophysin-positive nerve terminals ameliorating neuronal damage, mild microglia activation	[29, 179, 180]
Sprague-Dawley rats injected with rAAV-SNCA into SN	rh-SNCA	Reduction of SNCA inclusions in SN, induction of regulated T cells and activated microglia	[180, 339]
Two models: PDGF-SNCA mice expressing hSNCA under the PDGF-β promoter and mThy1-SNCA mice expressing hSNCA under the murine Thy1 promoter	C-terminus of SNCA (aa 110–130), also able to bind to full-length and N-terminal-truncated forms of α- syn, such as SNCA 96–140	Reduced SNCA oligomers in axons and synapses, reduced degeneration of striatal TH-immunoreactive fibers, clearance of SNCA involved microglia, improved motor and cognitive deficits in both models	[180, 340]
Mice expressing hSNCA under the control of the myelin basic protein promoter	Ag mimicking the C-terminus of SNCA or the original C-terminus peptide (aa 110–130)	Decreased accumulation of SNCA, reduced demyelination in neocortex, striatum and corpus callosum, reduced neurodegeneration, activation and clearance of SNCA by microglia, reduced spreading of SNCA to astrocytes	[341]
Passive immunization			
Tg-mice expressing hSNCA under the PDGF-β promoter, D-line	SNCA C-terminus Ab-9E4 (IgG1), epitope 118–126	Reduction of calpain-cleaved SNCA in neurons, no difference in microglia activation between control and Ab-treated mice, less motor and cognitive impairment	[342]
Tg-mice expressing hSNCA under the PDGF-β promoter, M-line	SNCA C-terminus Ab274 (IgG2a), epitope 120–140	Reduced accumulation of SNCA in neurons and astroglia, increased presence of SNCA in microglia, improved function in open field and pole tests	[180]
Tg-mice expressing hSNCA under the Thy-1 promoter, line 61	C-Terminus SNCA Ab: 1H7, 9E4, 5C1, and 5D12	Attenuated synaptic and axonal pathology in cortex, hippocampus and striatum, reduced accumulation of C-terminus-truncated SNCA in striatal axons and mitigated loss of TH fibers, reduced astrogliosis and microgliosis, improved motor and cognitive deficits	[343]
Tg-mice expressing hA30P SNCA under the Thy-1 promoter	SNCA protofibril-selective monoclonal Ab (mAb47)	Reductions of soluble and membrane-associated SNCA protofibrils in spinal cord, no change of astrocytic or microglial activity	
Mice overexpressing hSNCA under the PDGF-β promoter (line D)	Single-chain fragment variables against oligomeric SNCA fused to the low-density lipoprotein receptor-binding domain of APOE-B	Decreased oligomeric and phosphorylated SNCA accumulation in neocortex and hippocampus, reduced levels of astrocytes, improved memory function	[344]
Intrastriatal stereotaxic injections of SNCA preformed fibrils in wild type C57Bl6/C3H-mice	Monoclonal Ab: Syn303 (binds pathological conformations of human and mouse SNCA) targeting N-terminus)	Reduction of LB, amelioration of nigral DA-neuron loss, no differences in astrogliosis and microgliosis, improved motor behavior	[345]
Fisher 344 male rats injected into SN with rAAV expressing hSNCA	Ab against the N-terminal or central region of SNCA	Lowered levels of SNCA, reduced SNCA-induced DA-neuron loss, decreased number of activated microglia, partial improvement of behavioral deficits	[346]

### Surgical treatments

Most of the anti-PD drugs have several side-effects and are only transiently effective in a certain population of patients (Table 6). Additionally, they are unable to stop further DA-neuronal loss. Therefore, when there is no adequate relief after medication, clinicians resort to surgical treatments to reduce motor symptoms, especially during the advanced stages of PD. Currently, there are two commonly used surgical treatments for PD: (i) deep brain stimulation (DBS) and (ii) surgical lesions, such as a pallidotomy and/ or a thalamotomy [181].

**Fig. 14:**
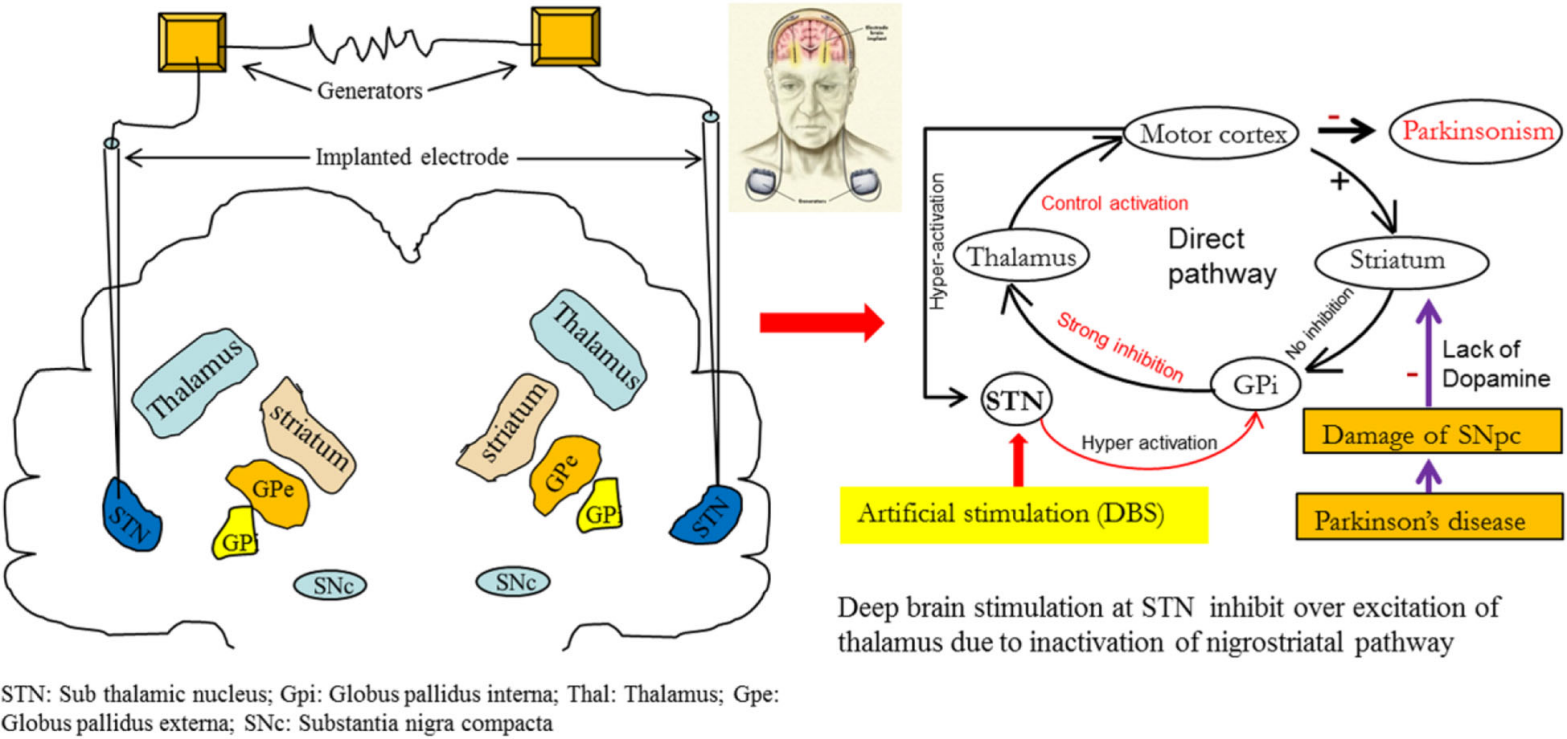
Schematic diagram show the process of DBS. In DBS, STN or thalamus or the globus pallidus interna (Gpi) (in this case STN) are stimulated by an implanted apparatus contains batteries that produce electrical stimulation (like a pace-maker). Stimulating the STN can activate the GPi, which can strongly inhibit the thalamus (right side circuitry) which can activate the motor cortex; in turn, allowing more control into the movement of limbs

**Fig. 15:**
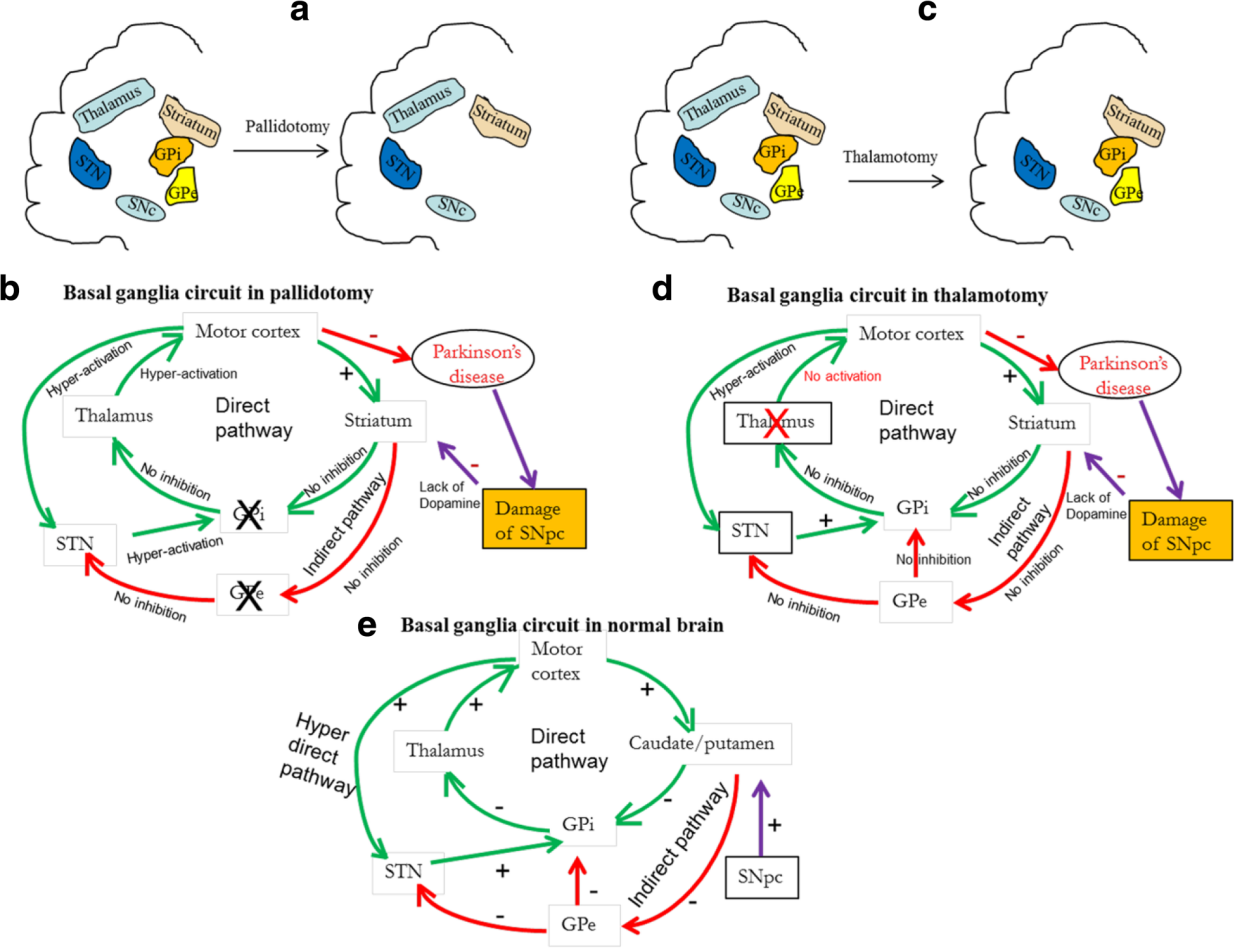
Schematic diagram showing pallidotomy (**a**), and thalamotomy (**c**) and the basal ganglia circuitory during pallidotomy (**b**) and thallatomy (**d**). In case of pallidotomy, the globus pallidus (GP) is surgically destroyed. In the case of a thalamotomy, both thalami are destroyed surgically, which causes a loos of thalamic excitation to the motor cortex, which can decrease Parkinson-like symptoms. Scematic diagram of basal ganglia circuitory in normal brain is shown in “**e**”

(i) *Deep brain stimulation (DBS).* Several basal ganglia nuclei become inactive or dysfunctional in PD. Surgical implantation of very fine electrodes in these areas can be used to keep them functionally active, a process called deep brain stimulation (DBS). The thalamus, globus pallidus interna (Gpi), or STN are target regions for DBS [182], where the electrodes are implanted, in one or both the hemispheres (Fig. 14).
  In DBS, devices containing two batteries, which generate finely tuned electrical currents for stimulating those deep brain areas, are implanted in both sides in the chest under the collar bone. The electrical pulses are generated by these batteries, which can be programmed precisely according to the specific needs of the PD patient. At 3–5 year’ intervals, the implanted batteries can be checked or replaced or recharged, accordingly. The DBS can reduce many primary motor symptoms of PD, and also decrease the need for L-DOPA to reduce dyskinesias [183]. In addition, the electrodes can be programmed to be turned on or off, as needed, by using a hand-held device [184]. However, the greatest disadvantage of using DBS, is that it requires surgical implantation of the device, which can cause potential complications, including stroke or hemorrhage, risk of infection, speech, or balance problems. Moreover, DBS is not effective in “atypical” parkinsonian syndromes, such as multiple system atrophy, progressive supra-nuclear palsy, or posttraumatic parkinsonism [184]. In addition, DBS is not used to treat the early stages or for treating mild symptoms of PD, or not suitable for treating the cognitive, psychological, or any other non-motor symptoms [183].
(ii) *Pallidotomy and thalamotomy.* The parts of the brain which control our voluntary movements include the globus pallidus (GP), a part of basal ganglia which has strong connections to the striatum and the thalamus. In pallidotomy, the surgeon selectively destroys a part of the GP (Fig. 15). Therefore, the synaptic connections with thalamus or striatum are altered in a way which decreases tremor, rigidity, bradykinesia, and posture abnormalities in PD patients [185].
  This surgical method can also reduce the amount of L-DOPA that the PD patient requires, which can decrease drug-induced dyskinesia and dystonia. Similarly, destruction of the thalamus, known as thalamotomy, can interrupt the connections between the basal ganglia and motor cortex, in ways that can restore neurotransmitter balance (e.g. glutamate excitation) and reduce symptoms, such as tremor [185]. Thalamotomy is used mainly for controlling tremor, and it is not very effective for bradykinesia, rigidity, or dyskinesias (Fig. 15).

### Cell transplantation therapy

**Fig. 16:**
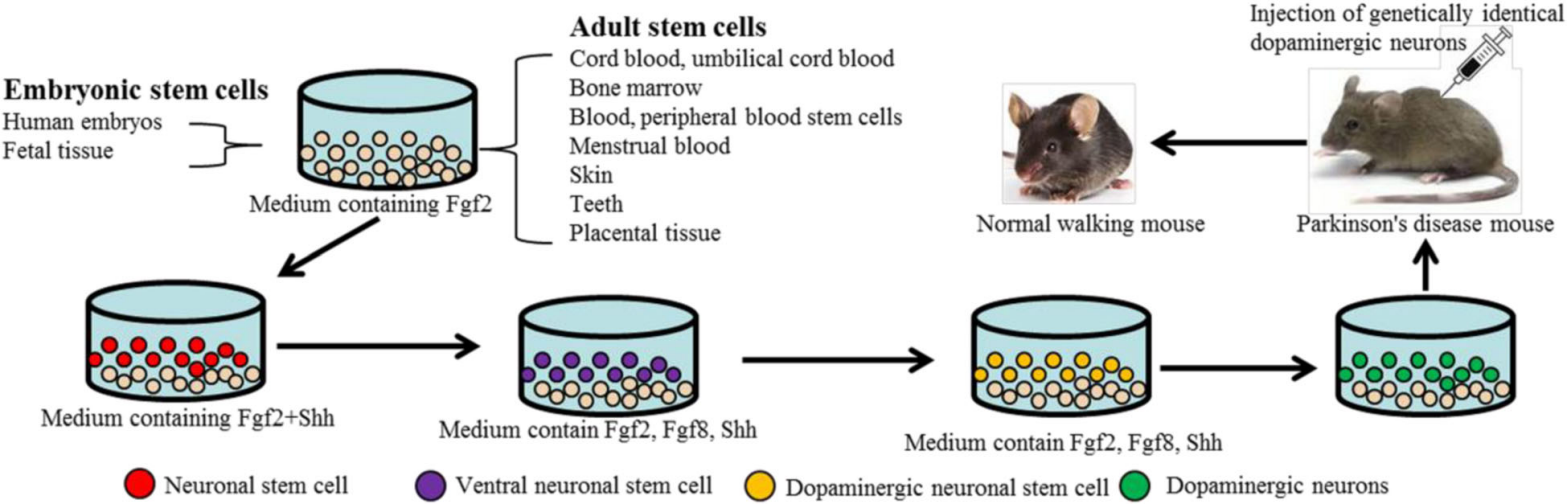
Different steps of generation of DA-neurons from stem cells for treating PD. Stem cells are obtained from different sources and converted to induced pluoripotent stem cells (iPSCs) using growth factors, such as Fgf2, Shh, Klf4 and c-Myc. The iPSCs is then converted to induced neuronal stem cells (iNSCs). These cells are then converted to DA-neurons by treating different growth factors. These DA-neurons are then injected to the brain of mouse model of PD to supply DA, which ultimately leads to the recovery of motor and cognitive deficits

Transplantation of neuronal stem cells into the brains of PD patients is considered to be one of the most promising approaches for treating this disease [186]. Over the last two decades, several investigators transplanted DA cells, such as adrenal medullary dopaminergic cells, into the striata in animal models of PD, [5, 6]. Recently, by manipulating several growth factors (such as FGF-2b, FGF8, SHH), researchers are able to generate DA-neurons from rodent embryonic stem cells (Fig. 16), and transplant them into the striata of animal models of PD. Interestingly, these transplanted neurons survive and integrate into the existing brain circuitry in animal model of PD and reverse the behavioral deficits [187, 188]. By over-expressing Nurr1, (a transcription factor for development of DA neuron) in embryonic stem cells, researchers can generate even more DA neurons [18, 19, 189, 190] to transplant into the brains of PD animals.

When human fetal-derived dopaminergic tissues are grafted into the striatum of the PD patients, an increase in DA levels is observed, suggesting that the implanted stem cells are able to survived and differentiate into DA neurons [191]. Further, when researchers implanted porcine-derived DA-producing cells into the brain of a PD patient, they observed modest clinical improvements, suggesting that DA-producing xenografts can survive in human brain [192]. Similarly, the greatest clinical benefits have been observed in allogeneic human fetal ventral mesencephalic (FVM) tissue transplantation in PD patient [193, 194]. These cells survive and make appropriate synaptic connections, while and increasing DA levels within the host cells. Similarly, high-quality DA-cells, such as porcine fetal mesencephalic cells, human retinal pigmented epithelial cells, and progenitor cells, have also been used for clinical or preclinical testing as a therapy for PD [190, 195]. However, further research is needed to evaluate the safety and efficacy of stem cell therapy before it can be approved as a treatment of human PD patients [196].

### Gene therapy for PD

Although most cases of PD are sporadic in nature, recent studies have confirmed that several genes are linked with the development of PD [16, 24, 197]. Therefore, gene therapy approaches may offer another promising means of treating PD [24].

**Fig. 17:**
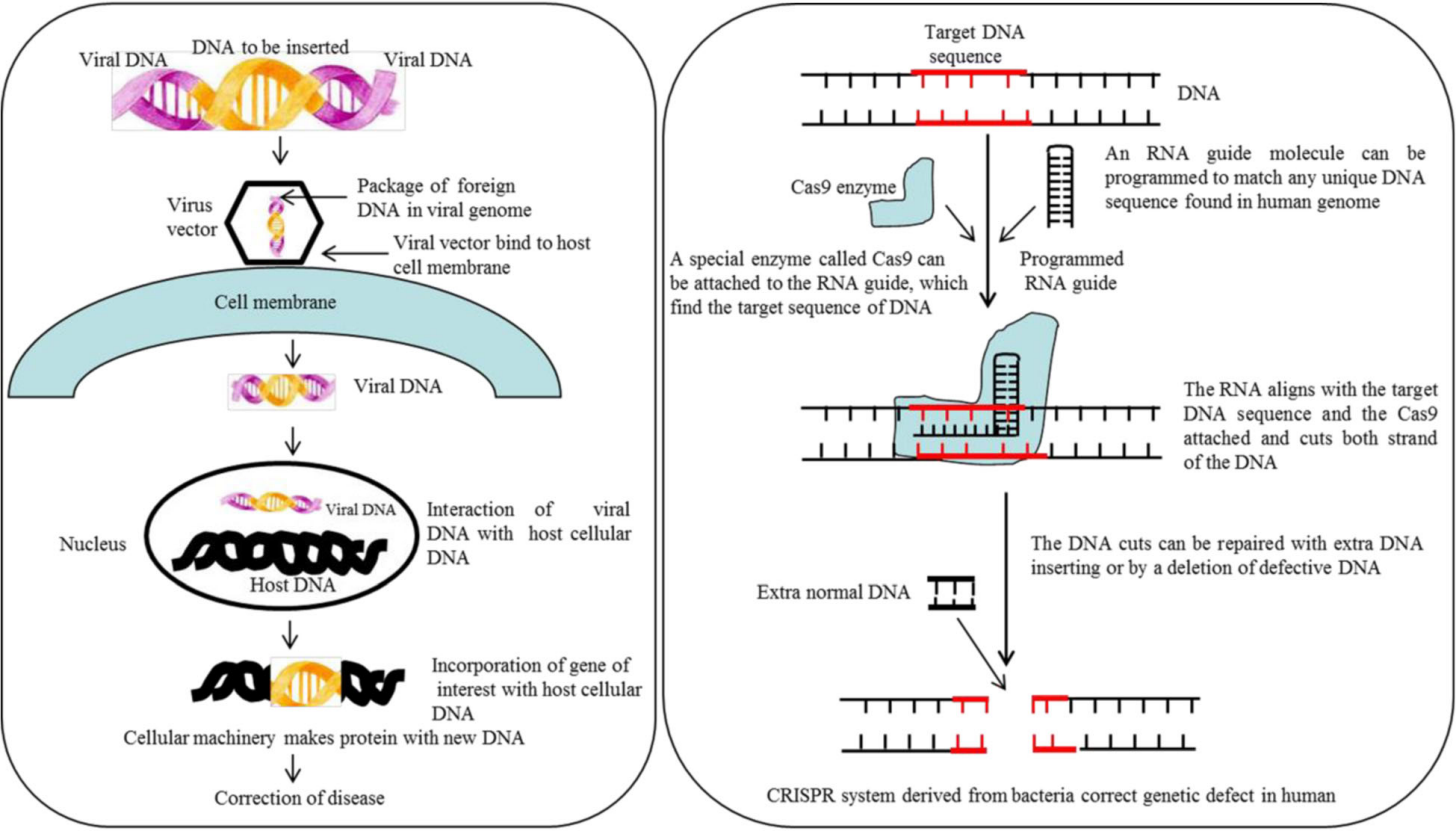
Schematic diagram of basics of rAAV-gene therapy. Left: The gene of interest is packaged within a rAAV vector. When the virus infects the host cell, it injects its DNA-containing gene of interest. This foreign DNA then crosses the nuclear membrane and binds with host DNA. Using protein machinery, the nucleus can make DNA and protein using the inserted DNA, replacing mutated or abnormal genes from host cell. Right: CRISPR-Cas9 system can be used to correct defect gene in PD and other genetic diseases. In presence of guide RNA (g-RNA) CRISPR-Cas9 enzyme can breakdown the DNA double strands in the locus where mutated or faulty genes are located. Then using DNA repair system, the normal DNA can be inserted in the cut site to get normal gene expression

(i) *Viral vectors-mediated gene delivery.* Currently, several viral vectors, such as lentivirus (LV), non-lentivirus, adeno-virus and recombinant adeno-associated virus (rAAV), containing target genes, are injected to the animal brain either by direct stereotaxic administration or via systemic injections. These viruses can integrate with the host cell and induce certain gene expression, promote DA-cell survival, as well as prevent degeneration of DA-neurons, which, ultimately, increases DA levels [24]. Similarly, the use of AAV2 to deliver gene for NGF, BDNF, GDNF, can increase regeneration of DA-neurons in SNpc and boost DA levels in striatum [198, 199]. For example, animals that were injected with the constitutively expressing GDNF vectors showed a long-term and stable improvement for GDNF levels [200]. Similarly, when AAV2-GDNF vectors were injected into the putamen of a MPTP-treated monkey, an enhancement of the locomotor activities and increased the DA-terminals were observed in the putamen [201]. Furthermore, delivering glutamic acid decarboxylase gene (GAD, the rate limiting enzyme for GABA synthesis) using an AAV (AAV-GAD) into the STN can increase GABA, which helps to balance the neuronal firing in the PD brain, resulting in a normalization of inhibitory signaling [202]. A clinical trial with AAV-GAD gene therapy into the STN has been shown to be safe and well tolerated by patients with advanced PD [203].
(ii) *AADC-TH-GCH Gene Therapy.* Chemical synthesis of DA from L-DOPA requires three-enzyme systems, involving aromatic amino acid decarboxylase (AADC), TH, and guanosine triphosphate cyclohydrolase (GTC). The TH and GCH catalyze the dietary tyrosine and convert it to L-DOPA, whereas AADC turns the L-DOPA to DA. Therefore, delivery of this triple gene therapy (AADC-TH-GCH) could be helpful to in maintaining basal DA levels in advanced PD [204] .
(iii) *RNA interference-based therapy.* Interference RNA (RNAi) is another powerful gene silencing approach, which could be used to inhibit *SNCA, PINK, or parkin* genes in PD. Recently, the polyethylene glycol-polyethyleneimine (PEG/PEI) siSNCA complex has been transfected into PC12 cells and a significantly decreased SNCA-mRNA expression, preventing MPTP-induced apoptosis [205].
(iv) *CRISPR-Cas9 gene editing system.* CRISPR/Cas-9 system is a powerful gene editing tool, including adding, disrupting, or changing the sequence of specific genes [21, 206, 207], which may be applied for PD-related gene therapy (Fig. 17). Using CRISPR/Cas9-mediated genome editing, Basu and colleagues developed a stable cell line that expresses SNCA tagged with a nano-Luc luciferase reporter. They observed an endogenous monitoring of SNCA transcription, which can make an efficient drug screening tool for therapeutic interventions in PD [208]. Although these gene therapy techniques look very promising, further studies are needed before their safe applications in PD patients can be initiated.

### Supplementation of neurotrophic factors

One of the primary reasons for neuronal cell death in PD is the depletion of neuronal growth factors, such as decrease BDNF, NGF, GDNF, and FGF-2b [199, 209]. It has been found that treatments with GDNF for 3–6 months can protect DA-neurons and promote their survival in animal models of PD [198]. Importantly, treatment with GDNF protects damaged DA neurons in the SNpc from their further degeneration in PD models [198]. Similarly, combined therapy of GDNF and neurotropin-4 on cultured DA neurons decreased oxidative stress and protected these neurons from further neurodegeneration [198]. Further, when BDNF was administered to rats in an aged rodent model of PD, an increase in the number of DA-receptors in the striatum was observed [210]. Other growth factors, such as FGF-2, released from activated astrocytes have also been shown to stimulate the survival of DA neurons [211], suggesting neurotrophins may be able to rescue DA-neurons in PD.

### Complementary and supportive therapies

To overcome some of the motor, as well as non-motor symptoms, several complementary and supportive therapies are available, including physical, occupational, and speech therapies.

(i) *Diet.* Diets containing fresh vegetables, fruits, green tea, and caffeine provide good sources of antioxidants, which may be beneficial for delaying or preventing oxidative stress-induced cell death in PD [212–214]. A high protein diet may aggravate the disease process, including an increase in constipation, and it may also reduce the effectiveness of L-DOPA [212]. Similarly, drinking plenty of liquids can reduce the chance of constipation. Several natural polyphenols (e.g. curcumin) can also prevent cell death in PD by interacting with amyloid proteins, including Aβ, p-tau, and SNCA [215, 216], as well as preventing further deterioration of DA-neurons [113, 216, 217]. Similarly, pleotropic actions of sodium benzoate, the principal ingredient of cinnamon also preserves DA neurons in the SNpc of animal models of PD [218, 219] **(**Table 8 **)**
(ii) *Aerobic exercise.* A plethora of evidence suggests that controlled aerobic exercise may improve gait, posture, mobility, and flexibility of PD patients [220, 221]. Sometimes, voluntary physical exercise, or physiotherapy, can strengthen muscle activity, decrease muscular tonicity and rigidity, improve balance, minimize gait problems, and strengthen certain muscles in PD patients [222]. Even in severe cases of the disease, regular walking, gardening, and other physical activities have been shown to be beneficial, and may improve the ability of the brain to increase DA synthesis. Moreover, experimental results indicate that daily aerobic physical exercise improves brain functioning by increasing neurogenesis, and secretion of neurotropic factors, such as BDNF, NGF, GDNF, FGF, which also help to maintain the emotional balance of people with PD [223].

**Table 8:** Some important dietary components which may prevent or delay PD progression

Diet	Chemical compound (s)	Usefulness in Parkinson’s disease	Refs.
Fava beans	Levodopa	Increases dopamine levels.	[347, 348]
Olive oil	Hydroxytyrosol	Antioxidants.	[212, 349, 350]
Turmeric powder	Curcuminoid	Antioxidants, decrease SNCA aggregation, anti-inflammatory	[215, 351, 352]
Cinnamon	Sodium benzoate	Stops the loss of Parkin and DJ-1 in Parkinson’s mice model.	[219, 218]
Soy (genistein)	Isoflavone	Increases dopamine, dopamine transporters, and Bcl_2_ levels	[212, 353]
Coffee	Caffeine	Antioxidants, adenosine A2A receptor antagonists.	[354–356]
Tea	Epigallocatechin-3-gallate (EGCG), theaflavins	Antioxidants, antiamyloid, decreases activity of COMT, increases dopamine uptake	[356–358]
Red wine	Resveratrol	Anti-amyloid, prevent blood clots and decreases inflammation.	[212, 359]
Fish	Omega-3 fatty acids (DHA)	Antidepressant effect, lowers blood pressure, and decreases the risk of strokes and inflammation.	[360–363]

### Rehabilitational therapy

Some of the primary symptoms of PD, especially gait, tremors, and rigidity can be reduced by the use of rehabilitational therapy [224]. Clinicians, who focus on treatments for non-motor symptoms, such as cognitive impairments, urinary tract dysfunction, sleep disorders, and micrographia, often utilize rehabilitational therapy [224]. For example, to reduce or to prevent muscle hypo-tonicity, PD patients need to be involved in their daily activities, such as moving from side to side, lifting their toes, breaking down their actions into individual steps, and keeping busy with house-hold work for at least few hours a day [225]. Similarly, walking, turning, or standing in different postures can help the patient maintain their balance and reduce the risk of falling. Patients with PD should take small steps when turning, but large, exaggerated steps when walking forward [225]. In addition, cognitive and other emotional dysfunction can be reduced by engaging in hobbies requiring focused attending, such as carpentry, fishing, playing cards, practicing “Yoga” or deep breathing and relaxation exercises [225]. These activities may help to control tremor, as well as reduce anxiety and depression. Speech therapy can help rectify the monotone voice and loss of volume which is often observed in PD patients, and it is also critical for evaluating and monitoring the ability to swallow [37].

## Monitoring PD progression

Using recent advanced and cutting-edge, noninvasive technology, the brains of PD patients can be more readily imaged for searching potential biomarkers or monitoring disease progression.

(i) *Neuroimaging.* The lack of easy access to the human brain is the major limitation for studying PD. Novel imaging techniques, such as PET biomarkers, including [18F]-DOPA can be used for estimating DA, [18F] dG for mitochondrial bioenergetics, [18F] BMS for mitochondrial complex-1, [11C] (R)-PK11195 for microglial activation. Similarly, SPECT imaging with 123Iflupane and βCIT can be used for measuring DAT, urinary salsolinol, and 8-hydroxy, 2-deoxyguanosine for neuronal loss [226, 227]. Similarly, terminal DOPA-decarboxylase (DDC) activity of PD brain can be measured with ^18^F–DOPA-PET, whereas the availability of presynaptic DAT can be assessed with tropane-based PET and SPECT tracers. Furthermore, vesicle monoamine transporter density in DA-terminals can be examined with ^11^C–dihydrotetrabenazine (DHTBZ)-PET [228]. Several investigators have used these techniques to study PD both in animals and human. For example, the striatal ^18^F–DOPA uptake has been shown to correlate with DA-neuronal numbers in the SNpc area of *post-mortem* PD brain and in MPTP-lesioned monkeys [229]. Similarly, ^18^F–DOPA-PET has been employed to study a series of asymptomatic heterozygote *parkin* mutation carriers and ^11^C–PK11195-PET has been used to study microglial activation in both the SNpc and pallidum of a PD patient [228]. In addition, brain regional N-acetyl-aspartate can be used for the in vivo assessment of neuronal loss in PD using MRS and MRI. Furthermore, fMRI and MRS can also be used to monitor the blood flow or metabolic status in the PD brain [230], and using fMRI, it is possible to detect PD and other synucleinopathies, such as LBD. These methods can also be helpful for studying the status of physiological activation of specific motor, cognitive, or mood swings after drug treatments.
(ii) *Biomarkers.* As PD is a disease with multiple etiologies and heterogeneous clinical symptoms, it is critical to develop several biomarkers to understand the disease more fully and to devise effective therapies. Although radioactive nucleotides are the most feasible biomarkers for screening of PD [231], fluid biomarkers, neuromelanin antibodies, candidate blood-based biomarker testing (e.g. SNCA, DJ-1, uric acid, epidermal growth factor, ApoA1) [232], gene expression profiling, metabolomics, protein profiling (e.g. Aβ and tau) and inflammatory markers (e.g. IL-6) from blood and CSF samples, can be used to predict PD. Because of the high cost of the procedures and the difficulty in administering these techniques, these methods are not always practical for screening PD patients in the general population [233]. In addition, investigating redox status and mitochondrial health could be used as early biomarkers for prediction of the onset of this disease [12]. Further, abnormal motor physiology, cognitive dysfunction, REM sleep disorders, autonomic dysfunction, loss of olfaction, and speech disturbances can, collectively be used to screen for PD [8]. Overall, expanding our knowledge of PD, combined with analyses of the pre-clinical and clinical symptoms, along with the development of more accurate biochemical and molecular markers, should translate into more effective and efficient ways to predict the onset and progression of this disease.

## Future directions for PD research and therapeutic developments

From the extensive animal research, we have addressed several critical questions in this complex field. However, no animal model is perfect in reproducing the behavioral deficits and neuropathological changes observed in human PD patients. Therefore, to mimic human PD symptoms and to address specific questions in this field several strategies can be taken, such as (i) development of a novel animal model, which can be produced by combination of two or more animal models, such as transgenic (for genetic effects), as well as sporadic (for toxin effects); (ii) using a range of non-DA drugs, including α2-adrenergic antagonists, serotonergic, and adenosine A2a antagonists, which may offer beneficial effects in late-stage developments of motor symptoms in PD; (iii) development of novel formulae for levodopa/carbidopa drugs (e.g. use of IPX066, XP21279, and Opicapone), MAO-inhibitors (e.g. use of safinamide:100–200 mg/day), which has an immediate and long-term clinical benefit on both early and advanced PD patients, without side effects, such as dyskinesia or depression; (iv) targeting ALP and UPS by novel pharmacological molecules may be an attractive strategy for PD therapy; (v) development of transplantation therapies using novel DA neurons from induced neuronal stem cells (iNSCs) or from induced pluripotent stem cells; (vi) micro-RNA or Si-RNA approach to inhibit mRNA of misfolded protein aggregates; (vii) application of novel gene editing techniques (e.g. CRISP-Cas9) for correction of mutated genes involved in PD. New drugs are constantly being developed tackle PD, and some of them are already improving quality of life in PD patients. There are several issues that need to be addressed before effective gene therapy can be safely used to treat PD. Some of these concerns involve the identification of the exact genes to be used for rectification, appropriate selection of novel vectors, development of safe marker genes, and modulators of precise gene expression in the CNS [24].

## Conclusions

The cases of PD are steadily increasing, due to the increase in the aged population. The financial and emotional impact of PD on public health and on those of the family and friends of those suffering from this disease is staggering. Therefore, prognosis or early detection will be critical for screening those who are susceptible for getting this disease. Several treatments are available, but none of them are particularly effective, in terms of reducing dopaminergic neuronal loss and restoring DA levels in the striatum. Moreover, some of the drugs have serious side-effects, and are expensive. Recently, scientists have introduced some promising alternative strategies, such as stem cell transplantation and gene therapy to treat this disease. However, most of these new treatment strategies are still under investigation, with most of these only being tested in animal models, so issues of safety and efficacy need to be adequately addressed before they can be used in clinical trials for PD. Nonetheless, the new therapeutic approaches described in this review offers significant hope that effective treatments for PD are in the near horizon.

## Abbreviations

Ach: Acetylcholine
ALP: Autophagy lysosomal pathway
AMPA: α-amino-3-hydroxy-5-methyl-4-isoxazolepropionic acid
Atg: Autophagy protein
BDNF: Brain derived neurotropic factor
CHIP: Carboxy-terminus of HSP70-interacting protein
CMA: Chaperone-mediated autophagy
COMT: Catechol-O-methyl transferase
COX-2: Cyclo-oxygenase-2
CRISPR: Cas9-clustered regularly-interspaced short palindromic repeats-CRISPR-associated protein 9
CSF: Cerebrospinal fluid
CT: Computarized tomography
DA: Dopamine
DAT: Dopamine transporter
DBS: Deep brain stimulation
FGF: Fibroblast growth factor
fMRI: Functional magnetic resonance imaging
FTDP: Frontotemporal dementia with parkinsonism
GABA: Gamma amino butyric acid
GAD: Glutamic acid decarboxylase
GDNF: Glial derived neurotropic factor
GI: Gastrointestinal
Gpi: globus pallidus interna
H_2_O_2_: Hydrogen peroxide
HSP: Heat shock protein
LAMP2A: Lysosome-associated membrane protein 2
L-DOPA: Levo-3,4-dihydroxyphenylalanine
LRRK2: Leucine-rich repeat kinase-2
LRRK2: l-leucine-rich repeat kinase 2
MAO-B: Monoamine oxidase-B
MPP: 1-methyl-4-phenylpyridinium
MPTP: 1-methyl-4-phenyl-1,2,3,6-tetrahydropyridine
MRI: Magnetic resonance image
MRS: Magnetic resonance spectroscopy
mtDNA: Mitochondrial DNA
NE: Norepinephrine
NFT: Neurofibrillary tangles
NGF: Nerve growth factor
NMDA: N-methyl-D-aspartate
OH: Hydroxyl radical
OHDA: Hydroxydopamine
PD: Parkinson’s disease
PET: Positron emission tomography
PFC: Prefrontal cortex
PINK-1: PTEN-induced putative kinase-1
PSF: Poly-pyrimidine tract-binding protein-associated splicing factor
PTEN: Phosphatase and tensin homolog
rAAV: Recombinant adeno-associated viral vectors
REM: Rapid eye movement
RNS: Reactive nitrogen species
ROS: Reactive oxygen species
SHH: Sonic hedgehog
SNpc: Substantia nigra pars compacta
SPECT: Single photon emission computed tomography
STN: Subthalamic nucleus
TH: Tyrosine hydroxylase gene
TPM: Two-photon microscopy
UCHL-1: Ubiquitin carboxyl-terminal esterase L-1
UPS: Ubiquitin-Proteasome System
VGCC: Voltage-gated calcium channel
β-CIT: 2β-carbomethoxy-3β-[4-iodophenyl)tropane -(4-iodophenyl)tropane]
